# New loci and coding variants confer risk for age-related macular degeneration in East Asians

**DOI:** 10.1038/ncomms7063

**Published:** 2015-01-28

**Authors:** Ching-Yu Cheng, Kenji Yamashiro, Li Jia Chen, Jeeyun Ahn, Lulin Huang, Lvzhen Huang, Chui Ming G. Cheung, Masahiro Miyake, Peter D. Cackett, Ian Y. Yeo, Augustinus Laude, Ranjana Mathur, Junxiong Pang, Kar Seng Sim, Adrian H. Koh, Peng Chen, Shu Yen Lee, Doric Wong, Choi Mun Chan, Boon Kwang Loh, Yaoyao Sun, Sonia Davila, Isao Nakata, Hideo Nakanishi, Yumiko Akagi-Kurashige, Norimoto Gotoh, Akitaka Tsujikawa, Fumihiko Matsuda, Keisuke Mori, Shin Yoneya, Yoichi Sakurada, Hiroyuki Iijima, Tomohiro Iida, Shigeru Honda, Timothy Yuk Yau Lai, Pancy Oi Sin Tam, Haoyu Chen, Shibo Tang, Xiaoyan Ding, Feng Wen, Fang Lu, Xiongze Zhang, Yi Shi, Peiquan Zhao, Bowen Zhao, Jinghong Sang, Bo Gong, Rajkumar Dorajoo, Jian-Min Yuan, Woon-Puay Koh, Rob M. van Dam, Yechiel Friedlander, Ying Lin, Martin L. Hibberd, Jia Nee Foo, Ningli Wang, Chang Hua Wong, Gavin S. Tan, Sang Jun Park, Mayuri Bhargava, Lingam Gopal, Thet Naing, Jiemin Liao, Peng Guan Ong, Paul Mitchell, Peng Zhou, Xuefeng Xie, Jinlong Liang, Junpu Mei, Xin Jin, Seang-Mei Saw, Mineo Ozaki, Takanori Mizoguchi, Yasuo Kurimoto, Se Joon Woo, Hum Chung, Hyeong-Gon Yu, Joo Young Shin, Dong Ho Park, In Taek Kim, Woohyok Chang, Min Sagong, Sang-Joon Lee, Hyun Woong Kim, Ji Eun Lee, Yi Li, Jianjun Liu, Yik Ying Teo, Chew Kiat Heng, Tock Han Lim, Suk-Kyun Yang, Kyuyoung Song, Eranga N. Vithana, Tin Aung, Jin Xin Bei, Yi Xin Zeng, E. Shyong Tai, Xiao Xin Li, Zhenglin Yang, Kyu-Hyung Park, Chi Pui Pang, Nagahisa Yoshimura, Tien Yin Wong, Chiea Chuen Khor

**Affiliations:** 1 Singapore Eye Research Institute, Singapore 169856, Singapore; 2 Duke-NUS Graduate Medical School, National University of Singapore, Singapore 169857, Singapore; 3 Department of Ophthalmology, National University of Singapore and National University Health System, Singapore 119228, Singapore; 4 Singapore National Eye Center, Singapore 168751, Singapore; 5 Department of Ophthalmology and Visual Sciences, Kyoto University Graduate School of Medicine, Kyoto 6068507, Japan; 6 Department of Ophthalmology and Visual Sciences, The Chinese University of Hong Kong, Hong Kong, China; 7 Department of Ophthalmology, Seoul Metropolitan Government Seoul National University Boramae Medical Center, Seoul 156-707, Korea; 8 Sichuan Provincial Key Laboratory for Human Disease Gene Study, Hospital of the University of Electronic Science and Technology of China and Sichuan Provincial People's Hospital, Chengdu 610072, China; 9 School of Medicine, University of Electronic Science and Technology of China, Chengdu 610072, China; 10 Key Laboratory of Vision Loss and Restoration, Ministry of Education of China, Beijing 100044, China; 11 Beijing Key Laboratory of Diagnosis and Therapy of Retinal and Choroid Diseases, Beijing 100871, China; 12 Department of Ophthalmology, People’s Hospital, Peking University, Beijing 100871, China; 13 Center for Genomic Medicine/Inserm U.852, Kyoto University Graduate School of Medicine, Kyoto 6068507, Japan; 14 Princess Alexandra Eye Pavilion, Edinburgh EH3 9HA, UK; 15 National Healthcare Group Eye Institute, Tan Tock Seng Hospital, Singapore 308433, Singapore; 16 Division of Human Genetics, Genome Institute of Singapore, Singapore 138672, Singapore; 17 Eye and Retinal Surgeons, Camden Medical Centre, Singapore 248649, Singapore; 18 Saw Swee Hock School of Public Health, National University of Singapore and National University Health System, Singapore 117549, Singapore; 19 Department of Ophthalmology, Saitama Medical University, Iruma 3500495, Japan; 20 Department of Ophthalmology, Faculty of Medicine, University of Yamanashi, Yamanashi 4093898, Japan; 21 Department of Ophthalmology, Tokyo Women’s Medical University Hospital, Tokyo 1628666, Japan; 22 Department of Surgery, Division of Ophthalmology, Kobe University Graduate School of Medicine, Kobe 6500017, Japan; 23 Shantou University/Chinese University of Hong Kong Joint Shantou International Eye Center, Shantou 515041, China; 24 Zhongshan Ophthalmic Center, Sun Yat-Sen University, Guangzhou 510060, China; 25 Aier School of Ophthalmology, Central South University, Changsha 410000, China; 26 Department of Ophthalmology, Xin Hua Hospital affiliated to Shanghai Jiao Tong University, School of Medicine, Shanghai 200025, China; 27 Beijing Tongren Eye Center, Beijing Tongren Hospital, Capital Medical University, Beijing Institute of Ophthalmology, Beijing 100730, China; 28 Cancer Control and Population Sciences, University of Pittsburgh Cancer Institute, Pittsburgh, Pennsylvania 15260, USA; 29 Department of Epidemiology, Graduate School of Public Health, University of Pittsburgh, Pittsburgh, Pennsylvania 15260, USA; 30 Hebrew University, School of Public Health, Jerusalem 91120, Israel; 31 Department of Ophthalmology, Seoul National University Bundang Hospital, Gyeonggi 463-707, Korea; 32 Department of Ophthalmology, University of Sydney and Westmead Millennium Institute, Sydney 2145, Australia; 33 Eye and ENT Hospital of Fudan University, Shanghai 200433, China; 34 BGI-Shenzhen, Shenzhen 518083, China; 35 Ozaki Eye Hospital, Miyazaki 8830066, Japan; 36 Mizoguchi Eye Hospital, Nagasaki 8570016, Japan; 37 Department of Ophthalmology, Kobe City General Hospital, Kobe 6500046, Japan; 38 Department of Ophthalmology, Seoul National University Hospital, Seoul National University College of Medicine, Seoul 110-744, Korea; 39 Department of Ophthalmology, School of Medicine, Kyungpook National University, Daegu 700-721, Korea; 40 Department of Ophthalmology, Yeungnam University College of Medicine, Daegu 705-802, Korea; 41 Department of Ophthalmology, College of Medicine, Kosin University, Pusan 606-701, Korea; 42 Department of Ophthalmology, Pusan Paik Hospital, Inje University College of Medicine, Pusan 614-735, Korea; 43 Department of Ophthalmology, Pusan National University Hospital, Pusan 602-739, Korea; 44 Medical Research Institute, Pusan National University, Pusan 602-739, Korea; 45 Department of Pediatrics, National University Health System and National University of Singapore, Singapore 119228, Singapore; 46 Department of Gastroenterology, Asan Medical Center and University of Ulsan College of Medicine, Seoul 138-736, Korea; 47 Department of Biochemistry and Molecular Biology, University of Ulsan College of Medicine, Seoul 138-736, Korea; 48 State Key Laboratory of Oncology in Southern China, Guangzhou 510060, China; 49 Department of Experimental Research, Sun Yat-Sen University Cancer Center, Guangzhou 510080, China; 50 Peking Union Medical College, Chinese Academy of Medical Science, Beijing 100730, China; 51 Department of Medicine, National University Health System and National University of Singapore, Singapore 119228, Singapore

## Abstract

Age-related macular degeneration (AMD) is a major cause of blindness, but presents differently in Europeans and Asians. Here, we perform a genome-wide and exome-wide association study on 2,119 patients with exudative AMD and 5,691 controls, with independent replication in 4,226 patients and 10,289 controls, all of East Asian descent, as part of The Genetics of AMD in Asians (GAMA) Consortium. We find a strong association between *CETP* Asp442Gly (rs2303790), an East Asian-specific mutation, and increased risk of AMD (odds ratio (OR)=1.70, *P*=5.60 × 10^−22^). The AMD risk allele (442Gly), known to protect from coronary heart disease, increases HDL cholesterol levels by 0.17 mmol l^−1^ (*P*=5.82 × 10^−21^) in East Asians (*n*=7,102). We also identify three novel AMD loci: *C6orf223* Ala231Ala (OR=0.78, *P=*6.19 × 10^−18^), *SLC44A4* Asp47Val (OR=1.27, *P*=1.08 × 10^−11^) and *FGD6* Gln257Arg (OR=0.87, *P=*2.85 × 10^−8^). Our findings suggest that some of the genetic loci conferring AMD susceptibility in East Asians are shared with Europeans, yet AMD in East Asians may also have a distinct genetic signature.

## Abstract (web summary)

Age-related macular degeneration (AMD) is a major cause of blindness worldwide. Here, the authors carry out a two-stage genome-wide association study for AMD and identify three new AMD risk loci, highlighting the shared and distinct genetic basis of the disease in East Asians and Europeans.

## Graphical abstract

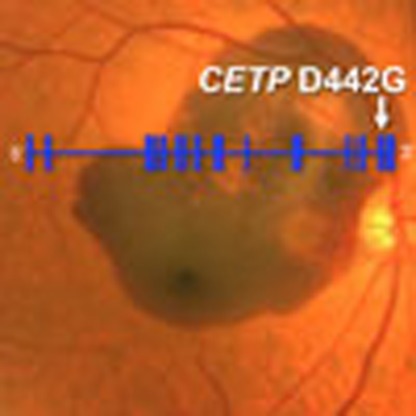

Age-related macular degeneration (AMD) is a progressive, blinding disease affecting millions of elderly individuals worldwide^1^,^2^. Several genome-wide association studies (GWAS) have identified common variants associated with AMD in European-ancestry populations^3^,^4^,^5^,^6^, and recently, rare genetic variation at *CFH*, *CFI*, *C3* and *C9* were also shown to strongly associate with AMD in Europeans^7^,^8^,^9^,^10^. However, there are few such studies in Asians^11^. Importantly, Asians appear to have a distinct clinical presentation of the disease (for example, absence of drusen and minimal fibrous scarring in polypoidal choroidal vasculopathy, a variant of AMD accounting for 20–55% of Asian patients with exudative AMD) and different responses to treatment (for example, poorer response to inhibitors of vascular endothelial growth factor (VEGF) compared with patients of European ancestry)^12^,^13^. It remains unclear whether there are differences in underlying genetic characteristics of AMD between patients of Asian versus European ancestry.

Concurrently, previous GWAS studies provide limited coverage of low-frequency coding variants, which may result in the loss of function and are often ethnic-specific. There is thus interest in genetic studies of AMD and other diseases beyond standard-content GWAS to discover potentially causative coding variants in different ethnic groups.

To address these questions, the Genetics of AMD in Asians Consortium perform a genome-wide (GWAS) and exome-wide association study (EWAS) of advanced AMD solely on the exudative (neovascular) disease subtype in East Asians. Compared with standard-content GWAS arrays, the exome array has significantly increased marker density across the coding human exome, thus increasing power to detect disease associations located within the coding frame. EWAS of AMD have not been previously conducted in either Europeans or Asians. In this paper, we present data from eight independent AMD case–control collections enrolled across multiple sites in East Asia, totalling 6,345 exudative AMD cases and 15,980 controls. This is the largest sample, to our knowledge, of East Asians ever assembled for genetic studies of AMD.

## Results

### Association with previously identified AMD variants

After genotype imputation, synchronization and stringent quality filters were performed, a total of 4,471,719 SNPs were assessed for the GWAS and 120,027 autosomal coding-frame SNPs for EWAS from 2,119 AMD cases and 5,691 controls (Table 1). Overall genomic inflation was very low (*λ*_gc_=1.031; Supplementary Fig. 1), suggesting minimal confounding of the disease association analysis by population stratification or other systematic study design biases. Data from the discovery stage analysis confirmed previously identified AMD variants in *ARMS2-HTRA1* rs10490924 (*P*=1.20 × 10^−103^), *CFH* rs10737680 (*P*=7.54 × 10^−38^), *CETP* rs3764261 (*P*=1.66 × 10^−12^), *ADAMTS9* rs6795735 (*P*=1.13 × 10^−5^), *C2*-*CFB* rs429608 (*P*=1.06 × 10^−4^), as well as *CFI* rs4698775 (*P*=7.5 × 10^−4^; Supplementary Table 1 and Supplementary Fig. 2). Our data also showed nominal evidence of replication in the same direction as the initial study for a further three previously reported variants (*TGFBR1* rs334353, *APOE* rs4420638, and *VEGFA* rs943080; *P*<0.05 for each). The remaining 8 out of 17 previously described SNPs that were non-monomorphic in our East Asian collections did not show evidence of replication in our study (Supplementary Table 1). A recently described rare, functional and highly penetrant genetic mutation within *CFI* (G119R, rs141853578) shown to confer markedly elevated risk of AMD in Europeans^14^ was observed to be non-polymorphic in our East Asian samples (Supplementary Table 1). Similarly, recently described rare mutations in *C3* (K155Q, rs147859257) and *C9* (P167S, rs34882957) were also shown to be non-polymorphic in our East Asian samples (Supplementary Table 1).

### Discovery of new SNP variants associated with AMD

Apart from verifying previous observations, our discovery analysis also revealed genome-wide significant association at *C6orf223* (rs2295334 encoding for A231A, *P*=1.41 × 10^−8^; Table 2, Supplementary Table 2 and Supplementary Fig. 2), a novel SNP marker not previously reported to associate with AMD risk. We observed a further 21 independent SNPs from distinct loci not previously implicated with susceptibility to AMD showing evidence of association surpassing *P*<1 × 10^−4^. We then brought forward all the 22 markers (Table 2) for replication genotyping in independent sample collections comprising 4,226 exudative AMD cases and 10,289 controls (Table 1). Replication evidence was compelling for *CETP* rs2303790 (encoding D442G; odds ratio (OR)=1.73, *P*=2.95 × 10^−16^), as well as for *C6orf223* rs2295334 (A231A; OR=0.80, *P*=5.25 × 10^−11^), *SLC44A4* rs12661281 (D47V; OR=1.22, *P*=5.13 × 10^−6^) and *FGD6* rs10507047 (Q257R; OR=0.88, *P*=7.69 × 10^−5^), leading to genome-wide significant findings in the meta-analysis of all 6,345 AMD cases and 15,980 controls (*P*<5.0 × 10^−8^ for each of the four loci; Table 2 and Supplementary Table 2). Genotyping clusters were directly visualized for the top SNPs and confirmed to be of good quality (Supplementary Fig. 3).

Of note, we did not observe any substantial difference in the association signals of the most significant SNPs in the subgroup analysis of our AMD cases by typical neovascular AMD (*n*=1,083 cases) and polypoidal choroidal vasculopathy (*n*=1,015 cases; Supplementary Table 3). The effect size for each of the top SNPs was similar between the two AMD subgroups.

### Conditional analysis

**Figure 1:**
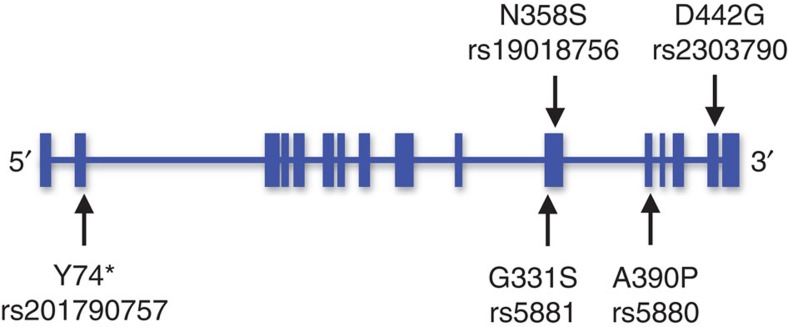
Genomic organization of the *CETP* gene. The position of five rare (minor allele frequency <0.05) amino-acid changes observed in the discovery samples are shown as indicated by the arrows. Horizontal bars represent the position of exons in the *CETP* gene.

The presence of the mutant *CETP* 442G (rs2303790) allele is seen only in East Asians (for example, Chinese, Japanese and Koreans; minor allele frequency (MAF) <5% in our controls) and not in South Asians, Europeans or Africans. This mutation is independent from all previously described common, non-coding polymorphisms near the *CETP* locus (*r*^2^<0.1; Supplementary Fig. 4). Regional association analysis conditioning on other known common AMD variants in *CETP* confirmed the independence of D442G from other nearby common variants (Supplementary Tables 4 and 5). We also genotyped and assessed multiple, rare, protein-changing mutations at *CETP*, including Y74Stop, G331S, N358S and A390P (Fig. 1). None of them showed association with AMD (Table 3). Mutational load and haplotypic analysis considering all amino-acid changes within *CETP* confirmed that the D442G mutation drove all signals of association between *CETP* and AMD (Table 4).

**Figure 2:**
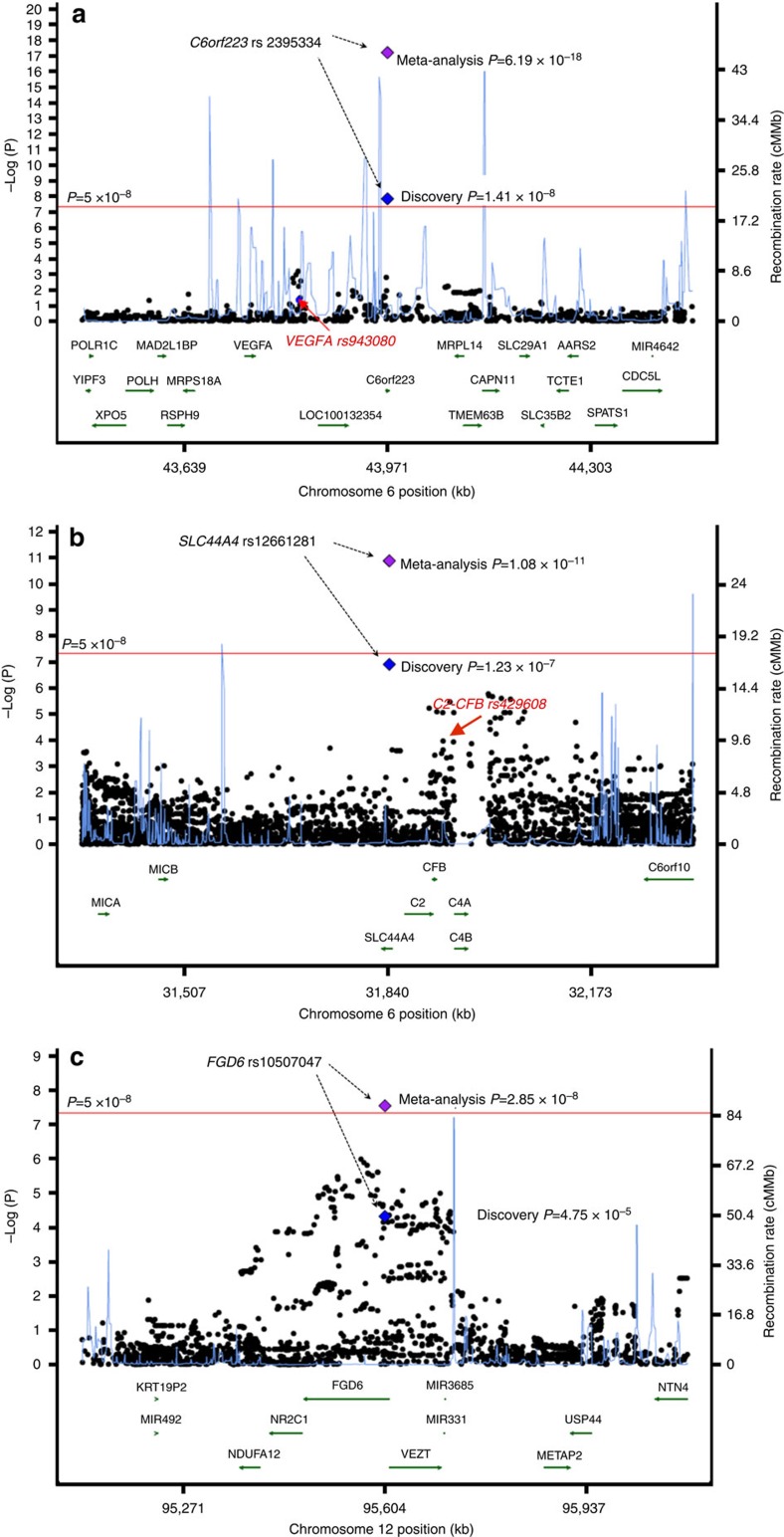
Regional association plots at the three risk loci for exudative age-related macular degeneration. (**a**) The *C6orf223* locus (rs2295334): *VEGFA* rs943080, which is located in the intergenic, non-coding region, was very strongly associated with AMD in Europeans, but this study of exudative AMD in East Asians could not reveal significant association with it (*P*=0.041 by score-based tests using logistic regression in 2,119 cases and 5,691 controls). The two markers are independent from each other (*r*^2^=0.0), as well as separated by strong recombination events in Asians. (**b**) The *SLC44A4* locus (rs12661281): *C2-CFB* rs429608 was very strongly associated with AMD in Europeans, but revealed modest evidence of association in our discovery stage (*P*=1.06 × 10^−4^). The two markers are independent from one another (*r*^2^=0.01). (**c**) The *FGD6* locus (rs10507047).

*C6orf223* is located ~220,000 base pairs downstream of *VEGFA* and ~150,000 base pairs from rs943080, a marker previously shown to strongly associate with AMD in Europeans^5^,^6^. In this study of AMD in East Asians, the evidence of association for *VEGFA* rs943080 was only nominally significant (*P*=0.041 in the discovery stage; Supplementary Table 1). Linkage disequilibrium analysis revealed no correlation between *C6orf223* rs2295334 and *VEGFA* rs943080 (*r*^2^=0.0), with both markers separated by significant recombination events (Fig. 2a). Logistic regression adjusting for the allele dosage at *VEGFA* rs943080 did not reveal any attenuation of the association signal for *C6orf223* rs2295334 (*P*=1.66 × 10^−8^; Supplementary Table 6). Regional association analysis including all markers from the genome and exome array data conditioning on *C6orf223* rs2295334 also did not reveal any secondary signal of association within its 1 Mb flanking region (Supplementary Table 7), thus pointing to *C6orf223* rs2295334 as a novel and uncharacterized genetic risk factor for exudative AMD in East Asians.

*SLC44A4* is located ~116,000 base pairs away from a previously reported AMD locus, *C2*-*CFB* (rs429608)^6^. Nevertheless, *SLC44A4* rs12661281 has no correlation with *C2-CFB* rs429608 (*r*^2^=0.01) and showed the strongest evidence of association with AMD within the genomic region (Fig. 2b), suggesting it to be also a new and uncharacterized risk factor for AMD. Logistic regression analysis adjusting for allele dosage at *C2-CFB rs429608* did not result in any significant change in magnitude of the association either at *SLC44A4* rs12661281, testifying to their mutual independence (OR_unconditioned_=1.38, *P*_unconditioned_=1.10 × 10^−7^; OR_conditioned for rs429608_=1.35, *P*_conditioned for rs429608_=1.49 × 10^−6^; Supplementary Table 8).

### Gene-based tests on mutational load

We next proceeded to conduct gene-based tests on mutational load to further investigate the role of low-frequency variants in exudative AMD for all the patient collections in the discovery stage. Gene-based tests are an alternative to single-marker tests for association, which are often underpowered to detect association with rare variants. We performed our tests as previously described^15^. To more directly address the impact of low-frequency, non-synonymous genetic variants, we considered only 109,296 such variants with MAF <5%. As a result, we were able to assess a total of 10,736 genes having at least two such variants using the sequence kernel association optimal (SKAT-O) test^16^. We did not detect significant evidence of association (*P*<5 × 10^−8^) between mutational load and AMD at any of the 10,736 genes tested, which are consistent across all three discovery sample collections. Nonetheless, while looking up on previously reported 22 genes within 17 distinct loci (Supplementary Table 1) associated with AMD in European populations, we note nominal evidence of association between genetic load at *CETP* (*P*_unconditioned_=5.38 × 10^−6^), whereby the association was almost entirely driven by D442G (*P*_conditioned for D442G_=0.96; Table 4) as well as *C2* and AMD (*P*=1.83 × 10^−6^, Supplementary Table 9). All the observations exceeding *P*<1 × 10^−4^ for gene-based tests on mutational load summarized across the three discovery collections are appended as Supplementary Table 9.

### CETP 442G, HDL and coronary heart disease

The mutant *CETP* 442G allele was shown to result in an abnormally functioning CETP protein^17^. As CETP is a critical component of the pathways that regulate high-density lipoprotein cholesterol (HDL-c)^18^, we assessed this mutation for associations with serum HDL-c levels using linear regression, with adjustment for age, gender and body mass index, in three population-based cohorts of Singaporean Chinese^19^,^20^,^21^ and Japanese^22^ (*n*=7,102, see details on the study cohorts in Supplementary Methods) where GWAS data were available. We noted a strong association between 442G allele and increased HDL-c levels (*β*=0.174 mmol l^−1^ per copy of 442G allele; reflecting an ~10% shift within the normal HDL range, *P*=5.82 × 10^−21^; Table 5). This effect size is at least twice that observed for other *CETP* variants reported in European populations (Supplementary Table 10)^23^,^24^.

Due to its strong effect on serum HDL-c, we assessed whether the mutant *CETP* 442G allele conferred any effect on individual susceptibility to coronary heart disease (CHD) in East Asians. Using 683 CHD cases and 1,281 controls from the Singapore Chinese Health Study (Supplementary Methods)^25^, we noted some degree of enrichment of the HDL-increasing, mutant 442G allele in the controls (2.89%) compared with the cases (2.42%, OR=0.83), although this did not reach statistical significance (*P*=0.39). However, our observations were consistent with a recent study from Japan which analysed *CETP* D442G in 4,399 CHD cases and 7,672 controls whereby enrichment of the 442G allele were also found in the controls (3.4%) compared with the CHD cases (2.8%; OR=0.83, *P*=0.02)^26^. We thus performed a meta-analysis of our study and the Japanese study, resulting in a consistent protective effect of this mutation with CHD (OR=0.83, *P*=0.011, *I*^2^=0.0%; Supplementary Fig. 5).

## Discussion

Our studies of exudative AMD in East Asians identified three novel loci (*C6orf233*, *SLC44A4* and *FGD6*), two of which (*SLC44A4* and *FGD6*) harbour coding, non-synonymous variants. These have not been identified in large samples of AMD patients of European descent^6^, thus validating the role of searching for coding variations in diverse ethnic groups to better understand mechanistic basis of complex diseases such as AMD.

Our most interesting findings were the identification of an uncommon East Asian-specific mutation at *CETP* (D442G) associated with exudative AMD. The association is the strongest (per-allele OR=1.70) observed outside of the classical *CFH* and *ARMS2*-*HTRA1* loci in East Asians. A common variant (rs3764261) mapping to the intergenic region between *HERPUD1* and *CETP* was previously linked to AMD in Europeans, yet its effect size was modest (OR=1.15)^5^ and independent from the D442G association. The mutant 442G allele is known to impair CETP function with reduction in plasma CETP mass and activity^17^,^27^, and is associated with elevated HDL-c in Japanese families^17^,^27^. This allele is absent in European populations^28^ and appears to be present only in East Asians, rendering it independent from all other previously described common, non-coding *CETP* polymorphisms. We showed that each copy of the dysfunctional 442G allele confers, on average, a rise in HDL-c levels of 0.174 mmol l^−1^ and confirmed findings from the earlier Japanese studies. Given the mean serum HDL-c concentration is 1.3 mmol l^−1^, this is a mutation of considerable effect size even from a population-based perspective. Notably, no other amino-acid substitutions within *CETP* detected by our GWAS and EWAS showed any evidence of association with AMD (Fig. 1 and Table 3), suggesting that the D442G mutation is a possible causative mutation of AMD in East Asians in the *CETP* locus. We are not surprised that *CETP* D442G did not show a clearly significant protective effect against CHD as increasing lines of evidence more directly implicate LDL as the driving force for CHD susceptibility^29^,^30^.

*C6orf223* is a newly mapped gene with yet unknown functional role. Its A231A synonymous coding change is fivefold rarer in Europeans (MAF=0.03) as compared with Asians (MAF >0.15, Supplementary Table 2). It is thus unsurprising that the European GWAS efforts have yet to detect this locus. *VEGFA* rs943080, a marker strongly associated with AMD in European-ancestry populations^5^, is in the vicinity of *C6orf223*, but its association with AMD is much weaker in this study of East Asians (Supplementary Tables 1 and 6), possibly reflecting the differences in therapeutic response to anti-VEGF treatment between Asians and Europeans^13^,^31^.

The D47V mutation within *SLC44A4* is not included in most of the routinely used genotyping arrays, but is now included as part of the exome array used in this study. The genomic region around the *SLC44A4* locus is more complex, being located within the broad MHC region on Chromosome 6 between *HLA* class I and class II genes. As this region is very polymorphic, and allele frequency differences between cases and controls could be confounded by even minor population stratification, we thus reassessed the associations with AMD by adjusting for the first 10 principal components (PCs) of genetic ancestry. We did not observe any change in the association signals observed from our standard analysis, which adjusted for the first five PCs (analysis adjusting for the top 10 PCs; OR=1.39, *P*=2.33 × 10^−7^ in the discovery phase), consistent with previous observations in Asian studies with well-replicated associations within the MHC region^32^,^33^. *SLC44A4* encodes for choline transporter protein-4, involved in sodium-dependent choline uptake by cholinergic neurons^34^. Defects in *SLC44A4* have been linked to sialidosis, which presents with a spectrum of symptoms including eye abnormalities^35^.

Prior studies on the basis of European patient collections have identified a total of four distinct AMD-associated loci on Chromosome 6 alone^6^. In this light, the burden of proof for the positive identification of *C6orf223* and *SLC44A4* (both are also located on Chromosome 6) is higher than usual due to the need for appropriate considerations of previously reported variants on the same chromosome. It is reassuring to note that both *C6orf223* and *SLC44A1* showed the strongest two signals of association with AMD outside of *CFH*, *ARMS2*-*HTRA1* and *CETP*. Our exhaustive analyses using logistic regression adjusting for allele dosages at previously described SNP markers suggest that both *C6orf223* and *SLC44A1* are unrelated to those previously reported, and thus they likely represent Asian-specific genetic associations for AMD.

Neither *FGD6* nor the genes within its vicinity (Fig. 2c) have ever been previously implicated in any ocular disorders. *FGD6* encodes for FYVE, RhoGEF and PH domain-containing protein 6, with its functions yet to be characterized. The Q257R mutation is less than half as common in Europeans (MAF=0.10) compared with East Asians (MAF=0.20–0.30), again possibly explaining the ability of our study to pick up this genetic effect.

The identified genes were expressed in human retinal pigment epithelium (Supplementary Table 11). Of the three non-synonymous substitutions, the *CETP* D442G variant was predicted by both PolyPhen^36^ and SIFT^37^ to likely be causing damage to the protein structure/function, the *FGD6* Q257R variant was predicted only by PolyPhen to be probably damaging but by SIFT to be tolerated and the *SLC44A4* D47V variant was predicted by both tools to be benign or tolerated. Although the use of both prediction algorithms has been reported to be moderately sensitive, they suffer from lack of specificity^38^, and thus more evidence should be sought with regards to the *FDG6* and *SLC44A4* non-synonymous variants.

Using HaploReg^39^, RegulomeDB^40^ and Encyclopaedia of DNA Elements (ENCODE)^41^ data, we identified variants within each of the four LD blocks in the 1000 Genomes Project (*r*^2^>0.8 and <250 kb from the top SNP) to apply functional annotations relevant to the regulation of transcription (Supplementary Table 12). In addition to their functions on amino-acid substitutions, all of the four identified variants lie within a DNase I hypersensitivity site or in a region where modification of histones is suggestive of promoter, enhancer and other regulatory activity, and/or have an influence on binding of transcription factors or effects on a specific regulatory motif. *C6orf223* A231A (rs2295334) and *FGD6* Q257R could tag genetic variants that lie in potential transcription-factor binding sites (Supplementary Table 13). Examination of a recently available large-scale eQTL mapping database^42^ indicates that out of the four novel genome-wide significant SNPs, markers *SLC44A4* D47V and *FDG6* Q257R could serve as cis-eQTLs. The minor allele at *SLC44A4* D47V (rs12661281) is associated with significantly altered expression of HSPA1B and CSKN2B, which are located within a 210,000 bp region flanking *SLC44A4* D47V. The minor allele at *FDG6* Q257R (rs10507047) is associated with significantly increased expression of the neighbouring *VEZT* gene (Supplementary Table 14). Given that these findings are based on expression in whole blood in European samples, further work will be needed to elucidate their role in retinal tissue and in Asian samples. Nonetheless, these could suggest possible alternate mechanisms whereby both non-synonymous substitutions potentially affect AMD risk apart from directly affecting the protein structure of their parent genes.

Our study examined mainly the exudative subtype of AMD, and therefore cannot be completely compared with other studies looking at advanced AMD including the choroidal neovascularization and geographic atrophy subtypes. We also note substantial differences in inter-ethnic MAF for most of the previously reported loci associated with AMD in European-ancestry populations (Supplementary Table 1). This could represent genuine differences in genetic architecture in AMD between Asians and Europeans, or that the low allele frequency in either ethnicity could result in insufficient power to replicate genome-wide significant hits initially observed in either Asians or Europeans. Overall, out of 21 previously reported SNPs showing strong evidence of association in Europeans, we were able to replicate 9 of them in our study of East Asians at *P*<0.05. Taken together with the three novel loci and one novel variant in *CETP* in East Asians discovered in this study, we postulate that the genetic mechanisms of AMD in Asians could, in part, be somewhat distinct from that in Europeans.

In summary, our genome-wide and exome-wide study of AMD provides new insights into the genetic mechanisms of AMD in East Asians. Our study highlights the value of searching for low-frequency, ethnic-specific genetic variants on the coding frame of AMD that may inform pathogenesis. Although some of the genetic loci conferring disease susceptibility in East Asians are shared with Europeans (for example, common variation mapping to *CFH*, *HTRA1* and *CETP*), we identified significant important differences in the fine-scale genetic architecture of AMD, which appear specific to East Asians. Such differences could underpin at least some of the inter-ethnic differences in clinical presentation and response to specific therapies, including the poorer response to anti-VEGF therapy in Asians.

## Methods

### Study design and phenotyping

We performed a GWAS and EWAS on neovascular AMD in East Asians. In the discovery stage, we included and genotyped 2,119 cases and 5,691 controls from three case–control studies from Singapore, Hong Kong and Japan. For replication, five independent case–control studies were conducted in Korea, Japan, and Guangdong, Sichuan and Beijing in China, totalling 4,226 cases and 10,289 controls. All the studies were performed with the approval of their local Medical Ethics Committee, and written informed consent was obtained from all the participants in accordance with the Declaration of Helsinki.

A detailed description of subject recruitment and phenotyping in each sample collection is provided in Supplementary Methods, and summarized in Table 1. In brief, the diagnosis of exudative AMD was made at each site by retinal specialists, according to standard clinical definitions on the basis of detailed ophthalmic examinations, including dilated fundus photography, fluorescein angiography, indocyanine green angiography and optical coherence tomography (Table 1). Grading of fluorescein angiograms for the presence of choroidal neovascularization were performed using a modification from the Macular Photocoagulation Study^43^. Indocyanine green angiography was performed to diagnose definitive polypoidal choroidal vasculopathy, a variant of AMD, using the Japanese Study Group guidelines^44^. Cases with other macular diseases such as central serous chorioretinopathy, high myopia and angioid streaks were excluded. Of the 2,119 exudative AMD cases included in the discovery phase, 1,083 (51%) were classified as ‘typical neovascular AMD’, 1,015 (48%) were polypoidal choroidal vasculopathy and 21 (1%) had one eye with typical neovascular AMD and the other eye with polypoidal choroidal vasculopathy. At each site, controls subjects without any clinical signs of AMD were either recruited from eye clinics or enrolled from population-based studies (Supplementary Methods).

### Genotyping and imputation

For the discovery stage, GWAS genotyping was performed using the Illumina Human OmniExpress or Human Hap610-Quad beadchips, and EWAS was done using HumanExome beadchips (Table 1). For replication, genotyping was performed using the MassArray platform (Sequenom), as well as using Taqman allelic discrimination probes (Applied Biosystems).

Stringent quality control filters were used to remove poorly performing samples and SNP markers in both the discovery and replication (de-novo genotyping) phases. For the GWAS, SNPs with a call rate of <95%, MAF of <1%, or showing deviation from Hardy–Weinberg Equilibrium (*P*<10^−6^) were removed from further statistical analysis. For the EWAS, SNPs with a call rate of <99%, MAF of <0.1% or showing deviation from equilibrium (*P*<10^−6^) were removed. The 99% threshold was used as many SNP markers on the exome array had MAF <5%, and as such, differential genotyping success rates between cases and controls as low as 2% could result in false-positive findings. SNPs which were not monomorphic (whereby at least one heterozygous carrier individual was present) were included for downstream analysis.

Routine quality control criteria on a per-sample basis were carried out, and poorly performing samples were removed from further analysis. The remaining samples were then subjected to biological relationship verification by using the principle of variability in allele sharing according to the degree of relationship. Identity-by-state information was derived using the PLINK software^45^. For those pairs of first-degree relatives so identified (for example, parent–offspring, full-siblings, as well as monozygous twins), we removed the sample with the lower call rate before performing PC analysis.

The imputation was carried out using IMPUTE2 version 2.2.2 with ASN population haplotypes from 1000 Genomes as reference, as described elsewhere^46^,^47^,^48^. Imputed genotypes were called with an impute probability threshold of 0.9 with all other genotypes classified as missing. Additional quality control filters were applied to remove SNPs with >1% missingness should the SNP have a MAF <5% in either cases or controls. For common SNPS with MAF >5%, the filtering criteria were set at >5% missingness.

### Statistical analysis

For the discovery stage, all exudative AMD cases and controls appear well matched when visualized spatially on PC analysis for each sample collection on a per-country basis for Hong Kong, Japan and Singapore and according to self-reported ethnicity (ethnic Chinese for Hong Kong and Singapore, and ethnic Japanese for Japan; Supplementary Fig. 6), using previously reported criteria^49^, indicating that population stratification is unlikely to confound the association results.

For both the discovery and replication stages, analysis of association with exudative AMD was carried out using 1-degree of freedom score-based tests using logistic regression. The tests model for a trend-per-copy effect of the minor allele on disease risk. For the discovery stage, we incorporated the top five PCs of genetic stratification into the logistic regression model to minimize the effect of residual population stratification^50^. We could not adjust for population stratification for the replication stage due to limited number of SNPs tested. Meta-analysis was conducted using inverse variance weights for each sample collection, which calculates an overall Z-statistic, corresponding *P* value and accompanying per-allele OR for each SNP analysed. Gene-based tests on mutational load was performed using the SKAT-O test^16^. The association between *CETP* D442G and serum HDL-c level was assessed using linear regression assuming an additive model of inheritance as previously described^23^ (due to serum HDL-c being distributed normally), with adjustment for age, gender and body mass index.

Regional association and PC plots were analysed and plotted using the R statistical software package.

### Power calculations

For the discovery stage (2,119 AMD cases and 5,691 controls), power calculations^51^ indicated that there is 80% power of detecting loci at *P*<1 × 10^−4^ (the threshold of association for bringing forward SNPs to the replication stage) at MAF as low as 10% with per-allele OR of 1.30. For rarer variants of higher penetrance, the discovery stage has 80% power of detecting loci at *P*<1 × 10^−4^ at MAF as low as 2% if the per-allele OR is at least 1.70. The entire sample (6,345 AMD cases and 15,980 controls) has 80% power to detect loci at *P*<5.0 × 10^−8^ at MAF as low as 2% if the per-allele OR is at least 1.55 or at MAF as low as 9% with per-allele OR of 1.25, in line with the effect sizes being reported in this study. Supplementary Table 15A shows the power calculations to detect SNPs at the threshold of *P*<1 × 10^−4^ in the discovery stage for bringing forward to the replication stage. Supplementary Table 15B shows the formal power calculations in the context of the combined discovery and replication stages.

## Author contributions

C.-Y.C., K.Y., L.J.C., J.A., K.-H.P., C.P.P., N.Y., T.Y.W. and C.C.K. designed the study. C.-Y.C., C.M.G.C., M.M., P.D.C., I.Y.Y., A.L., R.M., A.H.K., S.Y.L., D.W., C.M.G.C., B.K.L., Y.S., H.N., Y.A.-K., N.G., A.T., K.M., S.Y., Y.S., H.I., T.I., S.H., T.Y.Y.L., H.C., S.T., X.D., F.W., P.Z., B.Z., J.S., J.-M.Y., W.P.K., R.M.v.D., Y.F., N.W., G.S.W.T., S.J.P., M.B., L.G., T.N., P.M., P.Z., S.-M.S., M.O., T.M., Y.K., S.J.W., H.C., H.-G.Y., J.Y.S., D.H.P., I.T.K., W.C., M.S., S.-J.L., H.W.K., J.E.L., C.K.H., T.H.L., S.-K.Y., T.A. and W.T.Y. gathered clinical data. J.P., S.D., I.N., Y.A.-K., F.M., P.O.S.T., F.L., X.Z., Y.S., B.G., R.D., Y.L., M.L.H., J.N.F., C.H.W., X.X., Jinlong Liang, J.M., X.J., Y.L., Jianjun Liu, K.S., E.N.V., J.X.B., Y.X.Z. and C.C.K. generated genetic data. C.-Y.C., K.Y., L.J.C., J.A., Lulin Huang, Lvzhen Huang, K.S.S., P.C., Jiemin Liao, P.G.O., Y.Y.T. and C.C.K. analysed the data. C.-Y.C., K.Y., L.J.C., J.A., Lulin Huang, Lvzhen Huang, E.S.T., X.X.L., Z.Y., K.H.P., C.P.P, N.Y., T.Y.W. and C.C.K. interpreted the data. C.-Y.C., K.Y., L.J.C., J.A., E.S.T., T.Y.W. and C.C.K. drafted the paper. All the authors contributed to revision of the paper.

## Additional information

**How to cite this article:** Cheng, C.-Y. *et al*. New loci and coding variants confer risk for age-related macular degeneration in East Asians. *Nat. Commun.* 6:6063 doi: 10.1038/ncomms7063 (2015).

## Supplementary Material

Supplementary Information Supplementary Figures 1-6, Supplementary Tables 1-15, Supplementary Methods and Supplementary References

## Figures and Tables

**Table 1:** Baseline characteristics of exudative age-related macular degeneration cases and controls in the discovery and replication sample collections.

**Sample collection**	**Ethnicity**	No. of cases*	No. of controls*	**AMD phenotyping**	**Genotyping platform**
*Discovery*					
Singapore	Chinese	631	1,967	Dilated fundoscopy, FA, ICG & OCT	Illumina OmniExpress, 610 K & Exome Chips
Hong Kong	Chinese	507	2,967	Dilated fundoscopy, FA & ICG	Illumina OmniExpress & Exome Chips
Japan	Japanese	981	757	Dilated fundoscopy, FA, ICG & OCT	Illumina OmniExpress & Exome Chips
Subtotal		2,119	5,691		
					
*Replication*					
Korea	Korean	757	1,829	Dilated fundoscopy, FA, ICG & OCT	Sequenom MassArray & Taqman
Japan	Japanese	1,213	4,035	Dilated fundoscopy, FA, ICG & OCT	Sequenom MassArray & Taqman
Guangdong, China	Chinese	398	2,478	Dilated fundoscopy, FA & ICG	Sequenom MassArray & Taqman
Sichuan, China	Chinese	1,055	1,089	Dilated fundoscopy, FA & ICG	SNAPSHOT
Beijing, China	Chinese	803	858	Dilated fundoscopy, FA, ICG & OCT	Sequenom MassArray & Taqman
Subtotal		4,226	10,289		
All samples		6,345	15,980		

AMD, age-related macular degeneration; FA, fluorescein angiography; ICG, indocyanine green angiography; OCT, optical coherence tomography.

^*^No. of samples reflect those passing quality checks.

**Table 2:** Summary of results of the genome-wide and exome-wide association study on exudative age-related macular degeneration.

**SNP**	**Chr**	**Position**	**Nearest gene**	**Minor allele**	Discovery (2,119/5,691)*		Replication (4,226/10,289)*		Combined (6,345/15,980)*	
					**OR**	***P***_**discovery**_	**OR**	***P***_**replication**_	**OR**	***P***_**combined**_
*Loci reaching* P_*discovery*_*<1* × *10*^−*4*^ *and* P_*combined*_*<5* × *10*^−*8*^										
rs2303790	16	57,017,292	*CETP*	G	1.69	3.36 × 10^−7^	1.73	2.95 × 10^−16^	1.70	5.60 × 10^−22^†
rs2295334	6	43,970,827	*C6orf223*	A	0.75	1.41 × 10^−8^	0.80	5.25 × 10^−11^	0.78	6.19 × 10^−18^‡
rs12661281	6	31,842,598	*SLC44A4*	T	1.38	1.23 × 10^−7^	1.22	5.13 × 10^−6^	1.27	1.08 × 10^−11^§
rs10507047	12	95,604,290	*FGD6*	G	0.83	4.75 × 10^−5^	0.88	7.69 × 10^−5^	0.87	2.85 × 10^−8^†
										
*Loci reaching* P_*discovery*_*<1* × *10*^−*4*^ *but* P_*combined*_≥*5* × *10*^−*8*^										
rs62191056	2	227,779,676	*RHBDD1*	A	1.33	2.27 × 10^−5^	1.14	0.046	1.23	1.10 × 10^−5^
rs7274811	20	32,333,181	*ZNF341*	T	0.83	5.72 × 10^−5^	0.92	0.011	0.89	1.26 × 10^−5^
rs3894326	19	5,843,784	*FUT3*	T	0.75	6.22 × 10^−6^	0.91	0.27	0.81	1.85 × 10^−5^
rs117581914	19	9,236,724	*OR7G3*	G	2.02	3.27 × 10^−5^	1.36	0.19	1.78	3.05 × 10^−5^
rs2287921	19	49,228,272	*RASIP1*	C	1.74	7.70 × 10^−6^	1.20	0.13	1.43	3.56 × 10^−5^
rs17143419	7	70,829,578	*WBSCR17*	T	1.49	2.28 × 10^−5^	1.12	0.34	1.34	8.66 × 10^−5^
rs4280803	4	57,760,424	*REST*||	T	0.79	4.87 × 10^−5^	0.65	0.76	0.79	4.68 × 10^−5^
rs202018816	19	10,445,066	*ICAM3*	A	5.13	1.39 × 10^−5^	1.42	0.28	2.47	2.46 × 10^−4^
rs7165901	15	102,021,219	*PCSK6*	C	0.80	1.46 × 10^−5^	0.96	0.44	0.88	3.21 × 10^−4^
rs1891359	10	127,495,153	*UROS*	G	1.77	5.42 × 10^−5^	0.95	0.74	1.36	0.0044
rs215736	7	32,443,119	*PDE1C*-*LSM5*||	T	3.28	3.82 × 10^−5^	0.71	0.38	1.93	0.0049
rs2221338	2	68,224,745	*C1D*||	A	1.22	2.60 × 10^−5^	1.02	0.65	1.13	5.83 × 10^−4^
rs1538240	13	101,968,310	*NALCN*	T	1.24	2.92 × 10^−5^	1.03	0.59	1.14	6.54 × 10^−4^
rs7560053	2	228,220,769	*MFF*	T	0.83	7.00 × 10^−5^	0.98	0.76	0.90	0.0023
rs73509026	19	12,059,467	*ZNF700*	G	1.65	1.25 × 10^−5^	0.85	0.34	1.32	0.0039
rs1241050	4	141,410,099	*LOC152586*	T	0.72	3.35 × 10^−5^	1.02	0.79	0.86	0.0068
rs7612209	3	177,596,989	*AK056252*	A	1.21	6.45 × 10^−5^	0.93	0.11	1.06	0.087

Summary of SNPs that exceeded the threshold of *P*<1 × 10^−4^ on the basis of score-based tests using logistic regression in the discovery stage and were brought forward to the replication stage. In the combined meta-analysis of the discovery and replication samples, four new variants (the first four SNPs in the table) reached the threshold of genome-wide significance (*P*<5 × 10^−8^). All of the four new variants are coding variants (*CETP* rs2303790 encoding D442G; *C6orf223* rs2295334 encoding A231A; *SLC44A4* rs12661281 encoding D47V; and *FGD6* rs10507047 encoding Q257R). Physical positions and nearest genes are based on NCBI build 37 of the human genome.

SNP, single-nucleotide polymorphism; Chr, chromosome; OR, odds ratio for per copy of the minor allele.

^*^Number in parentheses presents the no. of cases and no. of controls, respectively.

^†^Heterogeneity *I*^2^=0% for *CETP* rs2303790 and *FGD6* rs10507047.

^‡^Heterogeneity *I*^2^=41.5%, random-effects *P* in the combined analysis=1.61 × 10^−13^.

^§^Heterogeneity *I*^2^=23.1%, random-effects *P* in the combined analysis=6.91 × 10^−9^.

^||^These index SNPs are located in intergenic regions. Other index SNPs are located within the genes.

**Table 3:** Single variant analysis (score-based tests using logistic regression) of the five observed *CETP* rare variants with exudative age-related macular degeneration in the discovery stage.

**Sample collections**	**SNP**	**Amino-acid change**	**A1**	**A2**	Genotype*		**MAF**		**OR**	***P*** **value**
					**Cases**	**Controls**	**Cases**	**Controls**		
Hong Kong	rs201790757	Y74*	C	A	0/0/507	0/3/2,962	0	0.0005	N/A	1
Hong Kong	rs5881	G331S	A	G	0/5/502	12/1/52	0.0049	0.0024	1.94	0.18
Japan	rs5881	G331S	A	G	0/3/970	0/4/759	0.0015	0.0026	0.59	0.49
Singapore	rs5881	G331S	A	G	0/5/635	0/21/1,935	0.0039	0.0054	0.73	0.52
Hong Kong	rs190187567	N358S	G	A	0/0/507	0/1/2,964	0	0.0002	N/A	1
Japan	rs190187567	N358S	G	A	0/1/972	0/0/763	0.0005	0	N/A	1
Hong Kong	rs5880	A390P	C	G	0/5/502	0/36/2,926	0.0049	0.0061	0.81	0.66
Singapore	rs5880	A390P	C	G	0/13/627	0/28/1,925	0.0102	0.0072	1.43	0.30
Hong Kong	rs2303790	D442G	G	A	4/45/458	2/172/2,791	0.0523	0.0297	1.80	2.8 × 10^−4^
Japan	rs2303790	D442G	G	A	4/84/885	0/41/722	0.0473	0.0269	1.79	0.0025
Singapore	rs2303790	D442G	G	A	1/44/595	0/98/1,858	0.0359	0.0251	1.48	0.034

SNP, single-nucleotide polymorphism; A1, minor allele; A2, major allele; MAF, minor allele frequency; OR, odds ratio for per copy of the minor allele.

^*^Data are number of genotypes A1A1/A1A2/A2A2.

**Table 4:** Results of association of the five observed *CETP* rare variants with exudative age-related macular degeneration using gene-based tests in 2,119 cases and 5,691 controls in the discovery stage.

**Gene**	**No. of variants**	**Variants (minor allele counts)**	Unconditional analysis*	Conditional analysis*
*CETP*	5	Tyr74*(3), Gly331Ser (56), Asn358Ser (2), Ala390Pro (81), Asp442Gly (536)	*P*=5.38 × 10^−6^	*P*=0.96

^*^Gene-based tests on mutational load (additive allele based using Burden tests) at *CETP*, unconditioned and conditioned for *CETP* Asp442Gly.

**Table 5:** Association of serum HDL cholesterol level and *CETP* D442G in East Asian population-based cohorts.

**Cohort**	**No. of samples**	**D442G genotype**	**Serum HDL cholesterol mean (s.d.)**, **mmol l**^−**1**^	**Association of HDL with D442G**				
				**Effect allele**	**Effect allele frequency**	Effect,* **mmol l**^−**1**^	**s.e.m.**	***P*** **value**
SCES	1,922	Gly/Gly	1.46†	442Gly	0.02	0.17	0.04	1.46 × 10^−6^
		Asp/Gly	1.49 (0.010)					
		Asp/Asp	1.32 (0.009)					
SP2-set1‡	1,044	Gly/Gly	—†	442Gly	0.03	0.17	0.04	5.38 × 10^−5^
		Asp/Gly	1.70 (0.014)					
		Asp/Asp	1.52 (0.011)					
SP2-set2§	888	Gly/Gly	—†	442Gly	0.03	0.17	0.05	3.46 × 10^−4^
		Asp/Gly	1.59 (0.012)					
		Asp/Asp	1.43 (0.012)					
Nagahama	3,248	Gly/Gly	2.32 (0.344)	442Gly	0.04	0.18	0.03	1.85 × 10^−9^
		Asp/Gly	1.83 (0.030)					
		Asp/Asp	1.66 (0.008)					
Meta-analysis	7,102			442Gly	0.03	0.174	0.018	5.82 × 10^−21^

SCES, Singapore Chinese Eye Study; SP2, Singapore Prospective Study Program; Nagahama, Nagahama Prospective Genome Cohort for the Comprehensive Human Bioscience.

^*^Effect per copy of the 442Gly allele on serum HDL-c in mmol l^−1^.

^†^Only one individual in SCES was homozygous (Gly/Gly) for the *CETP* 442G mutant allele. No Gly/Gly homozygotes were observed in both SP2 collections.

^‡^Genotyped on Illumina 610 K chip.

^§^Genotyped on Illumina 1 M chip.
