# Supplementary material for: New loci and coding variants confer risk for age-related macular degeneration in East Asians
Source: Nat Commun. 2015 Jan 28;6:6063. doi: 10.1038/ncomms7063 (PMC4317498; doi:10.1038/ncomms7063)
Supplement: Supplementary Information — Supplementary Figures 1-6, Supplementary Tables 1-15, Supplementary Methods and Supplementary References [file ncomms7063-s1.pdf]

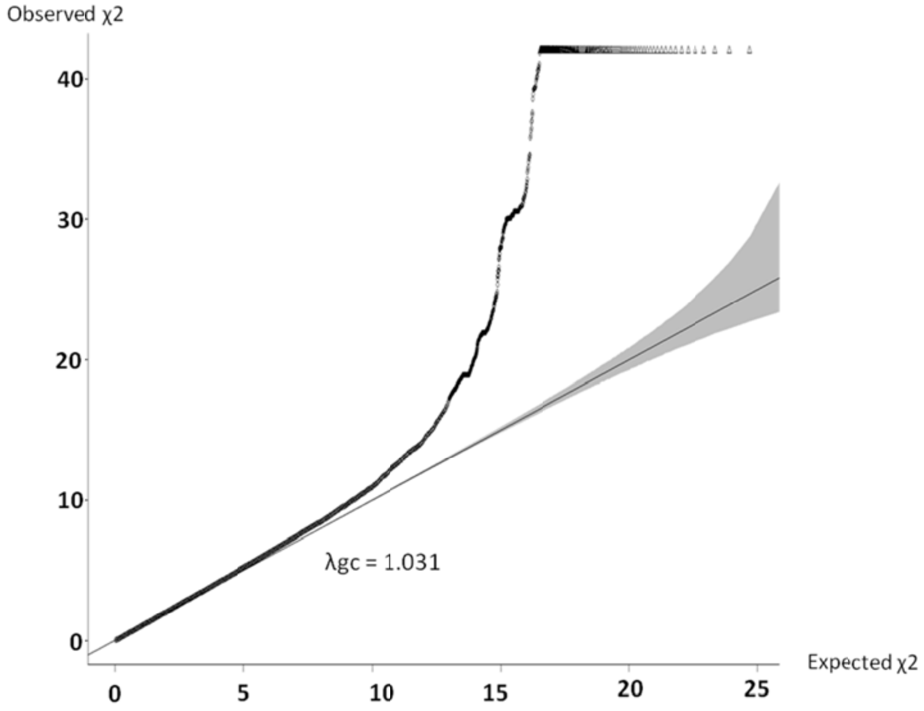

Supplementary Figure 1. Quantile-quantile plots of the tests statistics from the discovery analysis comprising 2,119 exudative AMD cases and 5,691 controls. The genomic inflation factor (λ_{gc}) was minimal at 1.031.

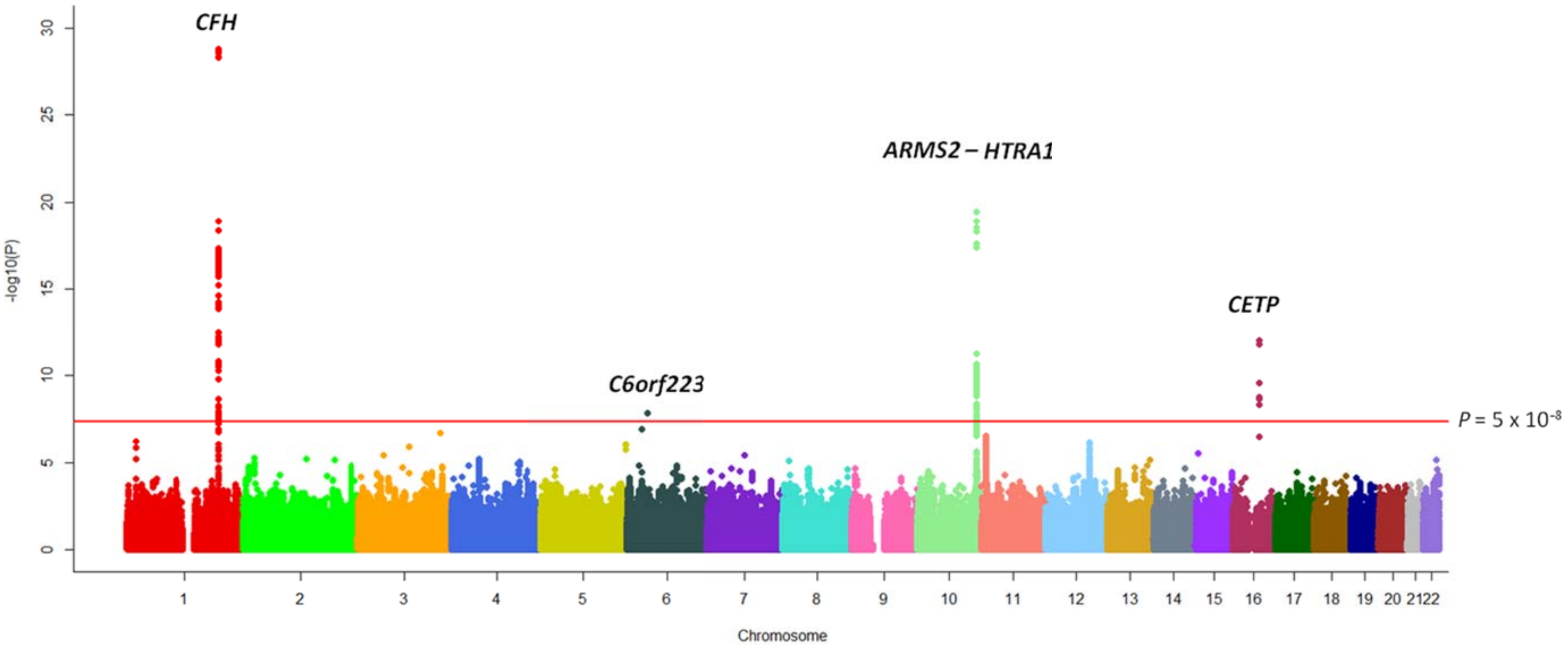

Supplementary Figure 2. Manhattan plot of the discovery stage analysis comprising 2,119 exudative AMD cases and 5,691 controls. Genome-wide significance was observed at SNPs mapping to the *CFH*, *ARMS2-HTRA1*, *CETP*, and *C6orf223* loci.

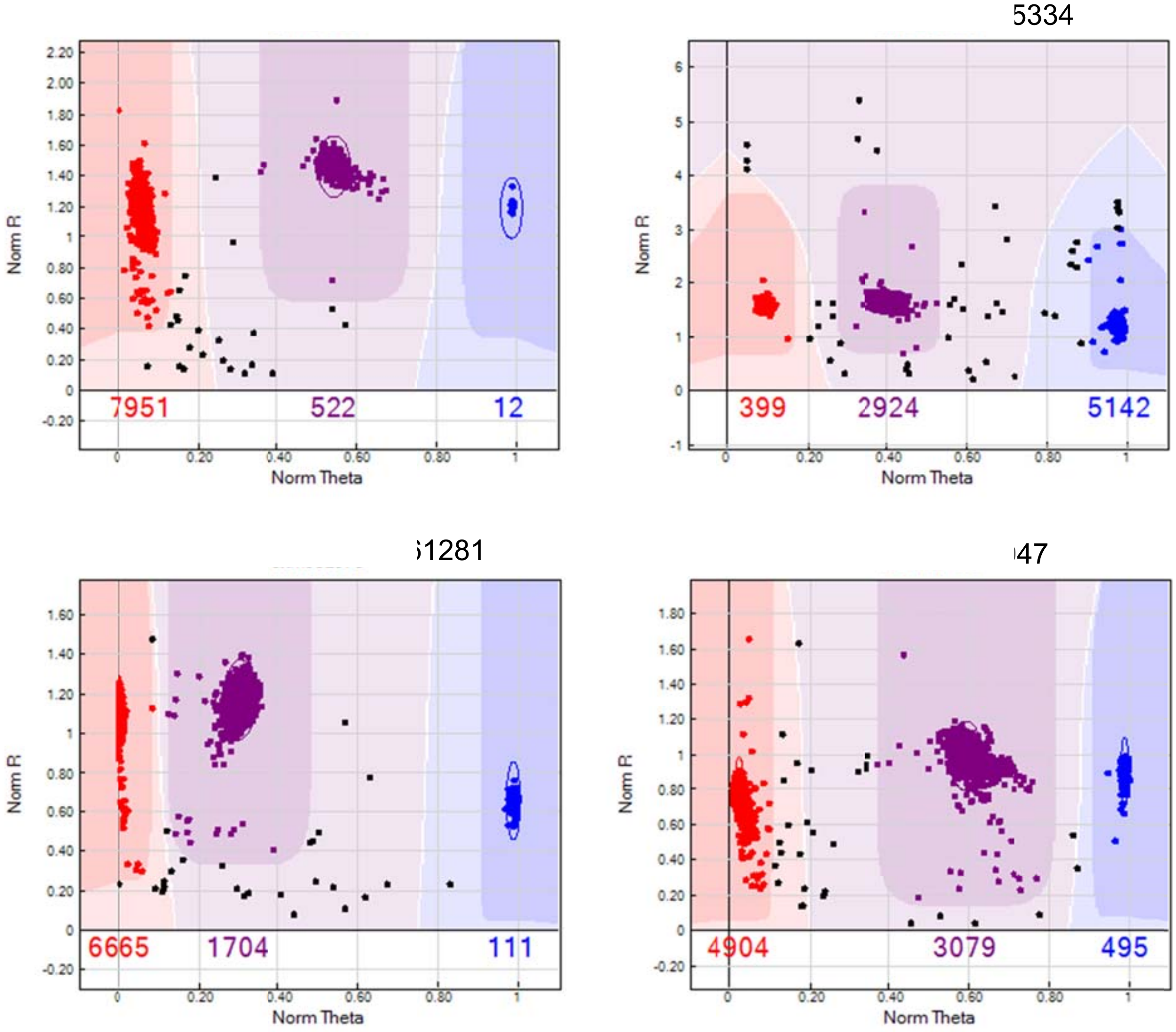

Supplementary Figure 3. Genotyping cluster plots for *CETP* D442G (rs2303790), *C6orf223* A231A (rs2295334), *SLC44A4* D47V (rs12661281), and *FGD6* Q257R (rs10507047)

a

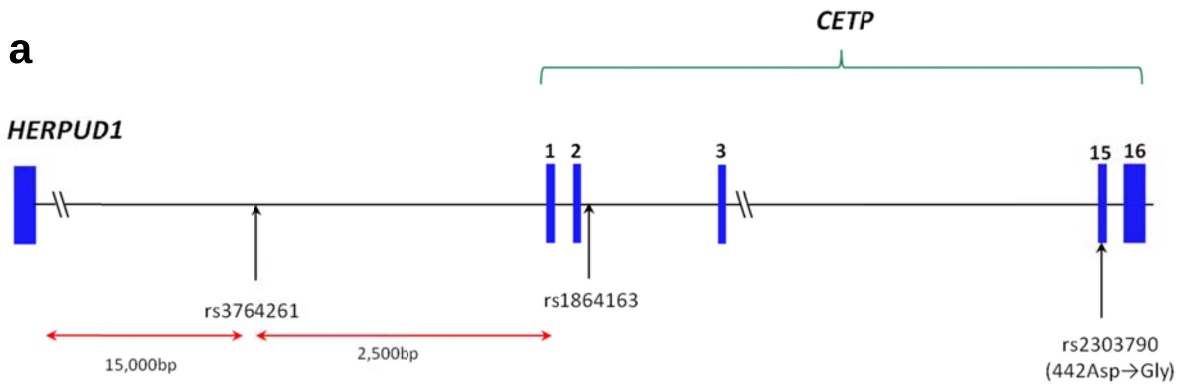

b

China	rs3764261	rs1864163	rs2303790
**rs3764261**	-	0.018	0.029
**rs1864163**	0.018	-	0.002
**rs2303790**	0.029	0.002	-

Japan	rs3764261	rs1864163	rs2303790
**rs3764261**	-	0.028	0.017
**rs1864163**	0.028	-	0.004
**rs2303790**	0.017	0.004	-

Hong Kong	rs3764261	rs1864163	rs2303790
**rs3764261**	-	0.018	0.089
**rs1864163**	0.018	-	0.005
**rs2303790**	0.089	0.005	-

Singapore	rs3764261	rs1864163	rs2303790
**rs3764261**	-	0.022	0.029
**rs1864163**	0.022	-	0.005
**rs2303790**	0.029	0.005	-

Korea	rs3764261	rs1864163	rs2303790
**rs3764261**	-	0.013	0.045
**rs1864163**	0.013	-	0.003
**rs2303790**	0.045	0.003	-

Supplementary Figure 4. Genomic location and linkage disequilibrium of the three markers within the *CETP* locus.

(**a**) Genomic location of the three markers within the *CETP* locus plotted with GWAS data available from the relevant collections as well as previously published data,[1, 2] and (**b**) pair-wise measures of linkage disequilibrium (LD) between these three *CETP* markers. Each square denotes the r^2 coefficient between the relevant genetic markers. Typically, $r^2 > 0.8$ denotes strong LD, $0.2 < r^2 < 0.8$ suggests moderate LD, and $r^2 < 0.20$ suggests weak LD. Markers rs3764261 and rs1864163 have been previously reported by European studied on AMD.[3, 4] *CETP* rs2303790 is East Asian specific and not present in Europeans.

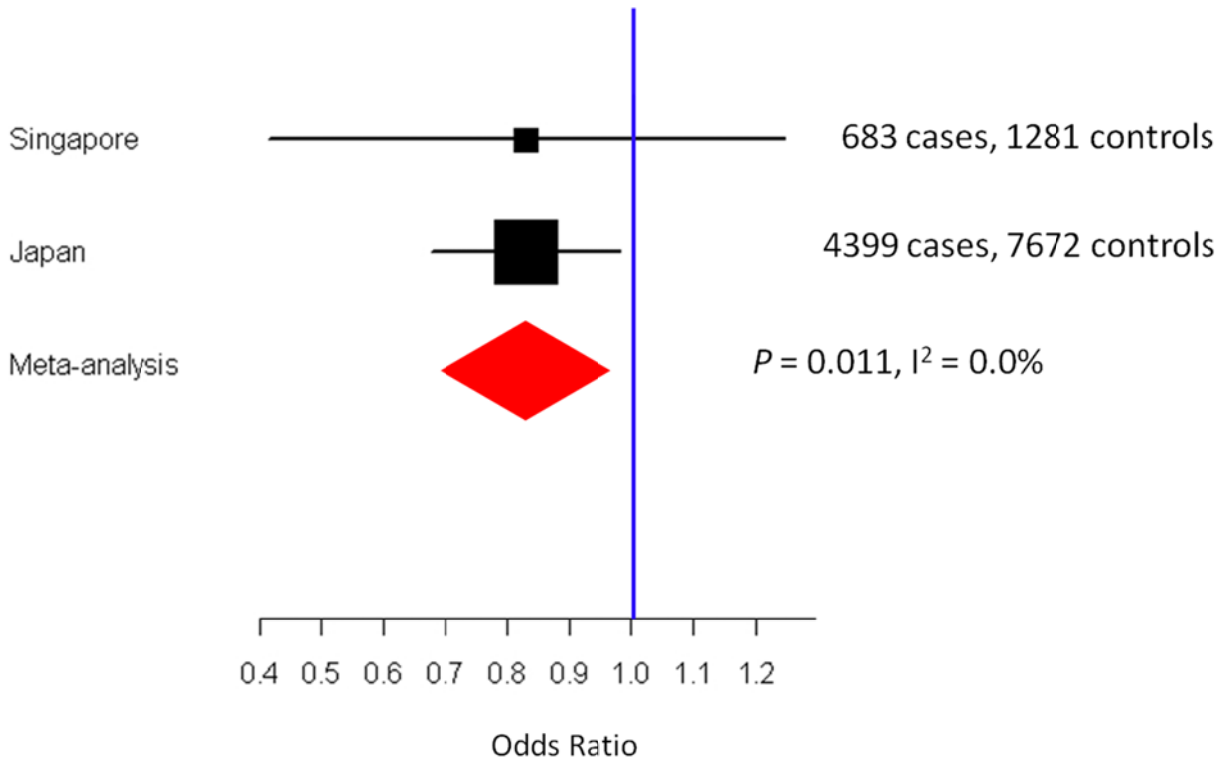

Supplementary Figure 5. Genetic association results between *CETP* D442G and coronary arterial disease in two independent sample collections

The Singaporean study (present) comprised 683 CHD cases and 1,281 controls. The Japanese study comprised 4,399 CHD cases and 7,672 controls.[5] The meta-analysis is performed using inverse-variance weights, summarizing each point estimate into Z-scores before meta-analysis.

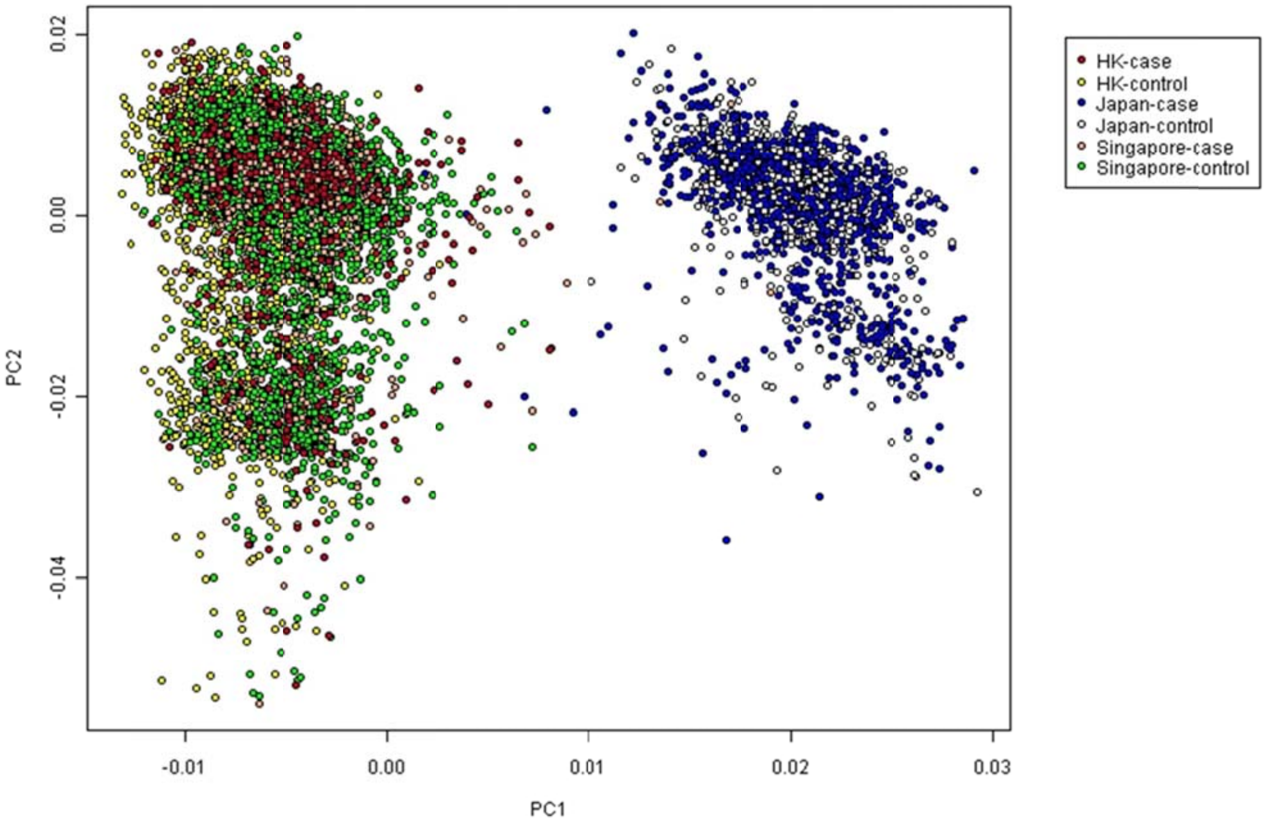

Supplementary Figure 6. Principal component (PC) analysis for the Hong Kong, Japanese, and Singaporean collections of exudative AMD patients and controls in discovery stage

PC analysis was undertaken to remove genetic outlier and to account for spurious associations resulting from ancestral differences of individual SNPs. For the discovery stage, all exudative AMD cases had genetically matched controls as visualized spatially on PC analysis for each sample collection, using previously reported criteria.[1] Shown here are the top two PCs.

Supplementary Table 1. Association results for previously reported AMD loci on SNPs directly genotyped by the integrated GWAS and EWAS in the discovery stage on 2,119 exudative AMD cases and 5,691 controls of East Asian descent in the current study

					Previous studies[3, 4, 6, 7, 8b]			Current study (discovery stage)			
**Locus name[a]**	**Chr**	**Position**	**SNP**	**Minor allele**	**MAF**	**p value**	**OR**	**MAF**	**p *value***	**OR**	**Power to detect[c]**
ARMS2-HTRA1	10	142.2 Mb	rs10490924	A	0.30	4×10^{-540}	2.76	0.47	1.20×10^{-103}	2.42	100
CFH	1	196.7 Mb	rs10737680	C	0.36	1×10^{-434}	0.41	0.39	7.54×10^{-38}	0.59	100
CETP	16	57.0 Mb	rs3764261	A	0.33	6.9×10^{-9}	1.15	0.17	1.66×10^{-12}	1.41	86.1
ADAMTS9	3	64.7 Mb	rs6795735	G	0.54	5×10^{-9}	0.91	0.25	1.13×10^{-5}	0.81	65.0
C2-CFB	6	31.9 Mb	rs429608	A	0.14	4×10^{-89}	0.57	0.07	1.06×10^{-4}	0.74	100
CFI	4	110.6 Mb	rs4698775	C	0.31	7×10^{-11}	1.14	0.20	7.50×10^{-4}	1.17	85.7
CFI	4	110.7 Mb	rs141853578[d]	A	<0.01	3.79×10^{-6}	22.2	0.00	-	-	0
TGFBR1	9	101.9 Mb	rs334353	C	0.27	3×10^{-11}	0.88	0.44	0.011	0.91	94.6
APOE	19	45.4 Mb	rs4420638	G	0.17	2×10^{-20}	0.77	0.10	0.026	0.86	99.7
VEGFA	6	43.8 Mb	rs943080	G	0.49	9×10^{-16}	0.87	0.24	0.041	0.91	92.9
IER3-DDR1	6	30.8 Mb	rs3130783	G	0.21	3×10^{-11}	0.86	0.10	0.063	1.12	74.1
COL10A1	6	116.4 Mb	rs3812111	A	0.36	2×10^{-8}	0.91	0.18	0.16	1.08	55.6
FRK-COL10A1	6	116.4 Mb	rs1999930	A	0.30	1.1×10^{-8}	0.87	<0.01	0.94	1.04	6.8
SLC16A8	22	38.5 Mb	rs8135665	A	0.21	2×10^{-11}	1.15	0.14	0.16	1.08	80.6
C3	19	6.7 Mb	rs2230199	C	0.20	1×10^{-41}	1.42	<0.01	0.19	2.45	12.1
C3	19	6.7 Mb	rs147859257[e]	C	<0.01	7.1×10^{-7}	3.13	0.00	-	-	0
LIPC	15	58.7 Mb	rs920915	G	0.52	3×10^{-11}	0.88	0.22	0.70	0.84	87.0
RAD51B	14	68.8 Mb	rs8017304	A	0.61	9×10^{-11}	1.11	0.45	0.82	0.99	82.7
COL8A1-FILIP1L	3	99.5 Mb	rs13081855	A	0.10	4×10^{-13}	1.23	0.04	0.998	1.0	64.1
C9	5	39.3 Mb	rs34882957[f]	A	<0.01	6.5×10^{-7}	2.2	0.00	-	-	0

Chr, chromosome; MAF, minor allele frequency; OR, odds ratio.

[a] A locus region is defined as a region flanking up to 250Kb of a genome-wide significant SNP.

[b] These previous studies include Yu Y et al., *Hum Mol Genet* 2011[3]; Fritsche LG et al., *Nat Genet* 2013[4]; Zhan X et al., *Nat Genet* 2013[6]; Seddon JM et al., *Nat Genet* 2013[7]; and Helgason H et al., *Nat Genet* 2013[8]. See the complete list in Supplementary References.

[c] Power to detect at $P < 0.05$ is expressed as a percentage. It is calculated based on minor allele frequency in Asians, and previously reported effect size in Europeans.

[d] Gly119Arg in *CFI*

[e] Lys155Gln in *C3*

[f] Pro167Ser in *C9*

Supplementary Table 2. Per-collection analysis for the four amino-acid substitutions showing genome-wide significant association with exudative AMD

SNP	Amino acid	Gene	Study	MAF case	MAF control	OR	p value
rs2303790	Asp442Gly	*CETP*	Hong Kong	0.052	0.030	1.80	0.00028
			Japan	0.047	0.027	1.79	0.0025
			Singapore	0.036	0.025	1.48	0.034
			**All discovery**			**1.69**	**3.36×10^{-7}**
			Sichuan, China	0.062	0.029	2.19	3.35×10^{-7}
			Beijing, China	0.037	0.025	1.48	0.051
			Guangdong, China	0.040	0.024	1.72	0.0074
			Japan	0.054	0.037	1.47	0.00029
			Korea	0.075	0.042	1.86	9.10×10^{-7}
			**All replication**			**1.73**	**2.95×10^{-16}**
			**All samples**			**1.70**	**5.60×10^{-22}**
rs2295334	Ala231Ala	*C6orf223*	Hong Kong	0.191	0.229	0.80	0.0089
			Japan	0.150	0.189	0.76	0.0033
			Singapore	0.201	0.258	0.71	2.80×10^{-5}
			**All discovery**			**0.75**	**1.41×10^{-8}**
			Sichuan, China	0.165	0.228	0.67	2.26×10^{-7}
			Beijing, China	0.207	0.249	0.79	0.0059
			Guangdong, China	0.216	0.244	0.85	0.088
			Japan	0.154	0.189	0.78	8.29×10^{-5}
			Korea	0.210	0.218	0.96	0.56
			**All replication**			**0.80**	**5.25×10^{-11}**
			**All samples**			**0.78**	**6.19×10^{-18}**
rs12661281	Asp47Val	*SLC44A4*	Hong Kong	0.120	0.095	1.12	0.32
			Japan	0.150	0.110	1.61	1.29×10^{-5}
			Singapore	0.140	0.110	1.45	0.00020
			**All discovery**			**1.38**	**1.23×10^{-7}**
			Sichuan, China	0.112	0.098	1.17	0.12
			Beijing, China	0.128	0.108	1.20	0.50
			Guangdong, China	0.141	0.111	1.31	0.016
			Japan	0.139	0.116	1.23	0.0018
			Korea	0.148	0.127	1.19	0.055
			**All replication**			**1.22**	**5.13×10^{-6}**
			**All samples**			**1.27**	**1.08×10^{-11}**

rs10507047	Gln257Arg	*FGD6*	Hong Kong	0.205	0.230	0.85	0.062
			Japan	0.307	0.345	0.85	0.022
			Singapore	0.182	0.221	0.79	0.0037
			**All discovery**			**0.83**	**4.75 x 10^{-5}**
			Sichuan, China	0.227	0.263	0.82	0.0052
			Beijing, China	0.218	0.234	0.91	0.25
			Guangdong, China	0.219	0.232	0.93	0.42
			Japan	0.289	0.312	0.90	0.032
			Korea	0.257	0.279	0.89	0.11
			**All replication**			**0.88**	**7.69 x 10^{-5}**
			**All data**			**0.87**	**2.85 x 10^{-8}**

MAF case, minor allele frequency in AMD cases; MAF control, minor allele frequency in controls; OR, odds ratio.

OR and *p*-values were derived after adjustment for the top 5 genetic principal components in the discovery stage.

Supplementary Table 3. Stratified analysis in the discovery samples for typical neovascular AMD and polypoidal choroidal vasculopathy

Gene	SNP	Collection	Sub-group	No. of cases/controls	OR	p value
SLC44A4	rs12661281	Hong Kong	tAMD	332/2967	1.19	0.17
SLC44A4	rs12661281	Japan	tAMD	418/757	1.46	0.0052
SLC44A4	rs12661281	Singapore	tAMD	333/1967	1.64	5.87×10^{-5}
		**All**	**tAMD**		**1.42 (1.23 - 1.65)**	**1.96×10^{-6}**
SLC44A4	rs12661281	Hong Kong	PCV	175/2967	0.95	0.80
SLC44A4	rs12661281	Japan	PCV	563/757	1.73	1.03×10^{-5}
SLC44A4	rs12661281	Singapore	PCV	277/1967	1.25	0.12
		**All**	**PCV**		**1.37 (1.17 - 1.62)**	**1.55×10^{-4}**
C6orf223	rs2295334	Hong Kong	tAMD	332/2967	0.85	0.13
C6orf223	rs2295334	Japan	tAMD	418/757	0.70	0.0033
C6orf223	rs2295334	Singapore	tAMD	333/1967	0.73	0.0027
		**All**	**tAMD**		**0.77 (0.68 - 0.87)**	**2.13×10^{-5}**
C6orf223	rs2295334	Hong Kong	PCV	175/2967	0.69	0.012
C6orf223	rs2295334	Japan	PCV	563/757	0.81	0.045
C6orf223	rs2295334	Singapore	PCV	277/1967	0.68	0.00099
		**All**	**PCV**		**0.74 (0.64 - 0.84)**	**8.42×10^{-6}**
CETP	rs2303790	Hong Kong	tAMD	332/2967	1.59	0.021
CETP	rs2303790	Japan	tAMD	418/757	1.92	0.0046
CETP	rs2303790	Singapore	tAMD	333/1967	1.57	0.047
		**All**	**tAMD**		**1.68 (1.31 - 2.15)**	**4.21×10^{-5}**
CETP	rs2303790	Hong Kong	PCV	175/2967	2.21	0.00073
CETP	rs2303790	Japan	PCV	563/757	1.74	0.010
CETP	rs2303790	Singapore	PCV	277/1967	1.38	0.21
		**All**	**PCV**		**1.77 (1.36 - 2.31)**	**2.47×10^{-5}**
FGD6	rs10507047	Hong Kong	tAMD	332/2967	0.87	0.16
FGD6	rs10507047	Japan	tAMD	418/757	0.84	0.065
FGD6	rs10507047	Singapore	tAMD	333/1967	0.81	0.044
		**All**	**tAMD**		**0.84 (0.75 - 0.94)**	**0.0025**
FGD6	rs10507047	Hong Kong	PCV	175/2967	0.83	0.19
FGD6	rs10507047	Japan	PCV	563/757	0.85	0.062
FGD6	rs10507047	Singapore	PCV	277/1967	0.78	0.033
		**All**	**PCV**		**0.83 (0.74 - 0.93)**	**0.0022**

tAMD, typical neovascular AMD; PCV, polypoidal choroidal vasculopathy; OR, odds ratio.

Supplementary Table 4. Association analysis between *CETP* rs2303790 (D442G) conditioned for *CETP* rs247616, *CETP* rs3764261, and *CETP* rs173539

SNP	Analysis conditioned on	Odds Ratio[a]	p value	r^2 *with* rs2303790
rs2303790	-	1.69	3.36×10^{-7}	-
rs2303790	rs247616	1.45	4.85×10^{-4}	0.052
rs2303790	rs3764261	1.45	4.31×10^{-4}	0.053
rs2303790	rs173539	1.53	4.64×10^{-5}	0.029

[a] Additive effect models

Supplementary Table 5. Regional association results at the *CETP* locus on Chromosome 16 before and after conditioning for rs2303790

Chr	BP	SNP ID	*P* before conditioning	*P*-conditioned on rs2303790	r^2 with rs2303790[a]	MAF[a]
16	57017292	rs2303790[a]	3.36×10^{-7}	-	-	3.2%
16	56989590	rs247616	9.32×10^{-13}	6.65×10^{-10}	0.052	17%
16	56993324	rs3764261	1.66×10^{-12}	1.14×10^{-9}	0.053	17%
16	56988044	rs173539	1.65×10^{-9}	1.18×10^{-7}	0.029	24%
16	57002732	rs9939224	0.0085	0.023	0.004	13%
16	56536660	rs150384293	0.012	0.014	0.0	3.3%
16	56933519	rs11643718	0.026	0.023	0.0	7.4%
16	56870627	exm1242557	0.029	0.026	0.0	0.9%
16	57073719	Exome_Asian_chr16-57073719-19	0.064	0.11		
16	56918023	rs139329616	0.068	0.082		
16	57091977	rs7206703	0.071	0.15		
16	56576763	rs9927912	0.073	0.10		
16	57047299	rs16965039	0.090	0.064		
16	57059372	Exome_Asian_chr16-57059372-19	0.11	0.10		
16	56519627	Exome_Asian_chr16-56519627-19	0.14	0.15		
16	56519604	Exome_Asian_chr16-56519604-19	0.17	0.15		
16	57006590	rs7499892	0.19	0.40		
16	57492176	rs78846023	0.19	0.16		
16	56500132	rs34883368	0.24	0.18		
16	57392733	rs4359426	0.25	0.21		
16	56545175	rs11373	0.30	0.24		
16	57075962	rs117840561	0.31	0.27		
16	56601720	rs666636	0.33	0.33		
16	56601722	rs666647	0.33	0.33		
16	57057619	rs1684575	0.36	0.48		
16	56602798	rs11643815	0.38	0.45		
16	56536654	Exome_Asian_chr16-56536654-19	0.38	0.44		
16	57016092	rs5882	0.43	0.083		
16	57074621	Exome_Asian_chr16-57074621-19	0.43	0.42		
16	57101340	rs7190199	0.45	0.51		
16	57101373	rs7185320	0.45	0.51		
16	56501176	exm2224962	0.47	0.49		
16	57140438	rs11863667	0.49	0.55		
16	57060353	rs28438857	0.50	0.40		
16	56969380	rs142638966	0.55	0.52		
16	57012012	rs5881	0.56	0.49		
16	57059427	rs74439742	0.58	0.63		
16	56985139	rs9989419	0.62	0.31		

16	56686886	Exome_Asian_chr16-56686886-19	0.64	0.62
16	56903674	rs146158333	0.66	0.59
16	57416775	Exome_Asian_chr16-57416775-19	0.69	0.65
16	57100453	rs149556424	0.70	0.67
16	57449687	rs34379253	0.71	0.73
16	56917989	Exome_Asian_chr16-56917989-19	0.72	0.76
16	57080528	rs289723	0.73	0.56
16	56969148	rs2217332	0.73	0.52
16	57068106	rs3995818	0.76	0.61
16	56913510	rs118121751	0.80	0.72
16	56545120	rs144680278	0.86	0.89
16	56500141	exm1861391	0.90	0.89
16	57015091	rs5880	0.93	0.94
16	57447890	Exome_Asian_chr16-57447890-19	0.95	0.99
16	56703807	rs112710021	0.96	0.94
16	57111699	exm2224992	0.97	0.97
16	57060689	Exome_Asian_chr16-57060689-19	0.99	0.96
16	56904556	Exome_Asian_chr16-56904556-19	1	1
16	57474687	rs11557674	1	1
16	57012094	exm1861738	1	1

Chr, chromosome; BP, genomic position Hg19 build; MAF, minor allele frequency.

[a]For SNPs showing p <0.05 with exudative AMD, minor allele frequency and pair-wise r^2 coefficient with rs2303790 is also provided.

Supplementary Table 6. Conditional logistic regression performed on both *C6orf223* rs2295334 and *VEGFA* rs943080

							Conditioned for			
					Before conditioning		*C6orf223* rs2295334		*VEGFA* rs943080	
Gene	Collection	SNP	MAF case	MAF control	OR	p Value	OR	p Value	OR	p Value
VEGFA	Singapore	rs943080	0.213	0.234	0.89	0.14	0.89	0.15	-	-
VEGFA	Japan	rs943080	0.308	0.333	0.90	0.15	0.90	0.16	-	-
VEGFA	Hong Kong	rs943080	0.212	0.221	0.96	0.58	0.95	0.58	-	-
	**All**				**0.91**	**0.041**	**0.92**	**0.049**	**-**	**-**
C6orf223	Singapore	rs2295334	0.201	0.258	0.71	2.80×10^{-5}	-	-	0.72	3.04×10^{-5}
C6orf223	Japan	rs2295334	0.150	0.189	0.76	0.0033	-	-	0.76	0.0037
C6orf223	Hong Kong	rs2295334	0.191	0.229	0.80	0.0089	-	-	0.80	0.0089
	**All**				**0.75**	$\mathbf{1.41 \times 10^{-8}}$	**-**	**-**	**0.75**	$\mathbf{1.66 \times 10^{-8}}$

MAF case, minor allele frequency in AMD cases; MAF control=minor allele frequency in controls; OR, odds ratio.

Supplementary Table 7. Regional association results at the *C6orf223* locus on Chromosome 6 before and after conditioning for rs2295334

CHR	BP	SNP ID	p before conditioning	p conditioned on rs2295334
6	43970827	rs2295334	1.41×10^{-8}	-
6	43968814	rs2295333	0.0016	0.18
6	43645833	rs3778492	0.018	0.018
6	43828582	rs4711751	0.032	0.039
6	43826627	rs943080	0.041	0.049
6	43581935	rs9333555	0.046	0.047
6	44221316	rs76772157	0.062	0.058
6	44222718	rs3734707	0.075	0.058
6	43748545	exm2244178	0.092	0.051
6	43646343	Exome_Asian_chr6-43646343-19	0.16	0.16
6	44145063	rs16871612	0.17	0.15
6	44253991	exm551532	0.18	0.18
6	43970658	rs115402675	0.23	0.089
6	43623326	rs2295947	0.24	0.21
6	43746655	Exome_Asian_chr6-43746655-19	0.24	0.23
6	44145138	Exome_Asian_chr6-44145138-19	0.27	0.32
6	43757896	rs998584	0.30	0.27
6	43638590	Exome_Asian_chr6-43638590-19	0.32	0.30
6	43582091	rs6941583	0.36	0.38
6	44255387	rs61742784	0.41	0.40
6	44115169	rs4714759	0.41	0.64
6	43806609	rs881858	0.42	0.47
6	43519108	Exome_Asian_chr6-43519108-19	0.44	0.50
6	44198362	rs45573936	0.47	0.47
6	43969839	rs80169011	0.48	0.99
6	43571679	rs147712217	0.48	0.55
6	43897727	rs9472155	0.48	0.47
6	44269873	rs139558578	0.49	0.45
6	43550096	rs61748656	0.52	0.58
6	44255455	Exome_Asian_chr6-44255455-19	0.52	0.57
6	43480045	exm2126895	0.53	0.50
6	44147881	Exome_Asian_chr6-44147881-19	0.55	0.45
6	44232920	rs2233434	0.56	0.50
6	43970487	exm550817	0.56	0.46
6	44107220	Exome_Asian_chr6-44107220-19	0.57	0.52
6	44291641	rs10948131	0.63	0.67
6	43488144	rs79222964	0.65	0.64
6	43581755	rs56307355	0.66	0.66

6	44255276	Exome_Asian_chr6-44255276-19	0.67	0.62
6	43581771	exm2127027	0.68	0.64
6	44310854	rs10948132	0.68	0.64
6	44140104	Exome_Asian_chr6-44140104-19	0.72	0.70
6	43968848	Exome_Asian_chr6-43968848-19	0.75	0.67
6	43639518	rs145782628	0.82	0.82
6	43758873	rs6905288	0.83	0.83
6	44255459	rs324146	0.86	0.82
6	44253765	rs2297336	0.87	0.82
6	43811762	rs9472138	0.88	0.78
6	43795968	rs943072	0.89	0.97
6	43785255	rs35349911	0.91	0.92
6	44147821	rs34710081	0.92	0.89
6	44224121	Exome_Asian_chr6-44224121-19	0.96	0.90
6	44141088	rs6938938	0.98	0.82
6	43541257	Exome_Asian_chr6-43541257-19	1.00	0.97
6	44272473	rs139280416	1	1

Chr, chromosome; BP, genomic position Hg19 build; MAF=minor allele frequency.

Supplementary Table 8. Conditional logistic regression performed on both *SLC44A4* rs12661281 and *C2-CFB* rs429608

Gene	Collection	SNP	MAF case	MAF control	Before conditioning		Conditioned for *C2-CFB* rs429608		Conditioned for *SLC44A4* rs12661281	
					OR	p value	OR	p value	OR	p value
SLC44A4	Singapore	rs12661281	0.14	0.11	1.45	1.98×10^{-4}	1.43	3.31×10^{-4}	-	-
SLC44A4	Japan	rs12661281	0.15	0.11	1.61	1.29×10^{-5}	1.54	1.05×10^{-4}	-	-
SLC44A4	Hong Kong	rs12661281	0.12	0.095	1.12	0.32	1.09	0.44	-	-
	**All**				**1.38**	$\mathbf{1.10 \times 10^{-7}}$	**1.35**	$\mathbf{1.49 \times 10^{-6}}$	**-**	**-**
C2-CFB	Singapore	rs429608	0.06	0.071	0.84	0.21	-	-	0.89	0.4
C2-CFB	Japan	rs429608	0.072	0.116	0.66	8.44×10^{-4}	-	-	0.71	0.008
C2-CFB	Hong Kong	rs429608	0.054	0.069	0.73	0.041	-	-	0.74	0.050
	**All**				**0.74**	$\mathbf{1.06 \times 10^{-4}}$	**-**	**-**	**0.78**	**0.0016**

MAF case, minor allele frequency in AMD cases; MAF control=minor allele frequency in controls; OR, odds ratio.

Supplementary Table 9. Results of gene-based tests on mutational load derived from the sequence kernel association optimal (SKAT-O) test. Shown here are the number of minor allele counts, and individual results for each of the three discovery collections (Hong Kong, Japan, and Singapore), as well as an overall meta-analysis p-value. Only Genes with $P_{SKATO\text{-}meta} < 1 \times 10^{-4}$ are shown.

	Singapore		Hong Kong		Japan		p value (SKAT-O tests)			
**Gene**	**# cases (n = 631)**	**# controls (n = 1967)**	**# cases (n = 507)**	**# controls (n = 2967)**	**# cases (n = 981)**	**# controls (n = 757)**	**Singapore**	**Hong Kong**	**Japan**	**Meta-analysis**
HSPA1L	13	17	9	20	50	33	0.0077	0.0045	0.057	1.05×10^{-6}
C9orf84	118	296	81	393	87	55	0.072	1.52×10^{-4}	0.70	1.05×10^{-6}
MOSPD3	11	29	16	24	15	9	0.94	1.23×10^{-12}	0.59	1.06×10^{-6}
C2	47	175	39	349	90	124	0.12	0.011	4.32×10^{-6}	1.83×10^{-6}
OR2AT4	8	9	8	13	12	6	0.031	0.0013	0.36	1.92×10^{-6}
ProSAPiP1	10	42	4	79	6	18	0.25	0.010	4.03×10^{-7}	4.37×10^{-6}
IGSF10	120	346	116	463	240	163	0.66	2.43×10^{-5}	0.15	4.52×10^{-6}
F13A1	14	19	10	46	114	54	2.90×10^{-4}	0.55	0.0028	4.69×10^{-6}
CETP	63	145	59	225	92	44	0.098	4.14×10^{-4}	0.0024	5.38×10^{-6}
ENDOV	36	91	37	135	51	29	0.0096	0.0031	0.13	9.26×10^{-6}
POLR3E	14	37	19	32	12	6	0.13	7.77×10^{-6}	0.36	1.18×10^{-5}
PCYOX1L	5	1	3	4	2	1	1.05×10^{-6}	0.051	0.82	1.68×10^{-5}
ZNF700	58	129	46	148	124	83	0.04	3.85×10^{-6}	0.49	4.47×10^{-5}
MYOM3	134	307	108	542	95	58	5.35×10^{-4}	0.073	0.23	8.16×10^{-5}

cases and # controls denote the number of minor allele counts in aggregate for all non-synonymous SNPs with individual minor allele frequency <0.05.

Supplementary Table 10. Association results between previously reported SNPs mapping near *CETP* and serum HDL-c levels in European-ancestry populations

SNP	Effect size,[a] mmol/L	p value	Sample size	Reference
rs173539	0.097	4×10^{-75}	19794	[9]
rs1800775	-0.067	3×10^{-13}	2758	[10]
rs1800775	-0.080	2×10^{-29}	5519	[10]
rs1800775	-0.062	1×10^{-23}	7940	[10]
rs1800775	-0.076	2×10^{-12}	5095	[10]

[a]Change in HDL (mmol/L) per copy of the effect allele.

Supplementary Table 11. Expression of the novel genes in adult human eye tissues

		PLEIR from The Ocular Tissue Database[a]				Eye SAGE[b]				
Gene	Probe ID	Retina	Choroid RPE	Sclera	Optic nerve	Macula	Macular RPE	Peripheral RPE	Peripheral retina B1[c]	RPE B1[c]
C6orf223	2908261	26.68	25.78	25.69	24.91	-	-	-	+	-
SLC44A4	2949488	27.68	30.69	32.79	35.32	-	+	+	-	-
FGD6	3466318	84.47	72.78	45.88	59.83	+	+	+	-	+
FGD6	3466369	22.67	29.94	26.09	36.27	+	+	+	-	+
CETP	3662417	25.97	30.07	28.72	26.53	-	-	-	-	+

RPE, retinal pigment epithelium.

[a]The gene expression in the Ocular Tissue Database (https://genome.uiowa.edu/otdb/) is indicated as Affymetrix Probe Logarithmic Intensity Error (PLIER) number. The PLIER numbers were calculated by GC-background correction, PLIER normalization, log transformation and *z*-score calculation.

[b]In the EyeSAGE datasets from NEIBank (http://neibank.nei.nih.gov/EyeSAGE/index.shtml), the gene expression is determined by tag counts in the Serial Analysis of Gene Expression (SAGE). We summarized all counts for each gene per tissue. '+' indicates the expression, and '-' labels no expression while the counts are 0.

[c]SAGE library prepared from the peripheral retina/RPE cells of a 88 year-old female patient.

Supplementary Table 12. Variants located within predicted regulatory regions and in LD ($r^2 > 0.8$) with the top SNPs in the four identified loci

Chr	Pos (hg19)	dbSNP ID	LD (r^2) with the top SNP	Reference allele	Alternate allele	ASN frequency	Variant Function	RefSeq genes	Promoter histone marks	Enhancer histone marks	DNAse	Proteins bound	Motifs changed
6	43961015	rs73733647	0.91	G	A	0.25		7.3kb 5' of C6orf223		K562, HMEC, HepG2			SREBP,TCF12,Zic
6	43963647	rs45584738	0.95	C	T	0.25		4.7kb 5' of C6orf223		NHLF	Fibrobl,Hepatocytes		
6	43963768	rs41282646	0.95	C	A	0.25		4.6kb 5' of C6orf223		NHLF	Hepatocytes		
6	43964680	rs76098842	0.96	TG	T	0.25		3.7kb 5' of C6orf223		4 cell types			6 altered motifs
**6**	**43970827**	**rs2295334**	**1**	**G**	**A**	**0.25**	**synonymous**	**C6orf223**	**6 cell types**	**HMEC**	**CD20+**	**CTCF,CFOS**	**9 altered motifs**
6	43972476	rs60012957	0.99	G	T	0.26	3'-UTR	C6orf223	H1	6 cell types	HMEC,Gliobla		
6	43972625	rs2273306	0.99	C	G	0.26	3'-UTR	C6orf223	H1	5 cell types	GM12878,8988T,HSMM_emb	EBF1	5 altered motifs
6	43972729	rs2273307	0.99	C	G	0.26	3'-UTR	C6orf223	H1	5 cell types	4 cell types	EBF1	5 altered motifs
6	43972733	rs2273308	0.99	C	T	0.26	3'-UTR	C6orf223	H1	5 cell types	4 cell types	EBF1	7 altered motifs
6	43972845	rs2273309	0.99	A	G	0.26	3'-UTR	C6orf223		4 cell types	9 cell types		En-1,Pax-3,Pax-6
6	43973461	rs9968846	0.99	A	G	0.26	3'-UTR	C6orf223		5 cell types	H1-hESC,pHTE	POL24H8	16 altered motifs
6	43973964	rs56118681	0.99	T	G	0.26		269bp 3' of C6orf223		5 cell types	LNCaP		7 altered motifs
6	43974116	rs59822205	0.99	G	C	0.26		421bp 3' of C6orf223		5 cell types		HNF4A	LF-A1,SP1
6	43974836	rs73422500	0.99	C	T	0.26		1.1kb 3' of C6orf223		4 cell types	H1-hESC	POL24H8,POL2B,POL2	T3R
6	43975750	rs73736909	0.85	G	A	0.24		2.1kb 3' of C6orf223		H1	H1-hESC,pHTE,HL-60		7 altered motifs
**6**	**31842598**	**rs12661281**	**1**	**T**	**A**	**0.12**	**missense**	**SLC44A4**		**K562, HepG2**			**LUN-1,p300**
12	95559600	rs10859842	0.87	T	A	0.26	intronic	FGD6		K562			7 altered motifs
12	95561195	rs10859843	0.85	T	C	0.26	intronic	FGD6			Fibrobl		AP-2rep,Rad21
12	95564940	rs7136350	0.85	A	G	0.26	intronic	FGD6					
12	95569637	rs11107925	0.86	G	C	0.26	intronic	FGD6		Huvec, K562			6 altered motifs
12	95574831	rs10777670	0.88	G	A	0.26	intronic	FGD6		K562			Hoxb8,TATA
12	95575951	rs11107928	0.93	C	A	0.25	intronic	FGD6		K562			Pou2f2
12	95577884	rs4128507	0.86	T	C	0.26	intronic	FGD6					4 altered motifs
12	95580818	rs12830425	0.93	T	G	0.25	intronic	FGD6		GM12878			HEY1,TATA
12	95583662	rs4762660	0.88	A	G	0.26	intronic	FGD6					Sox
12	95587797	rs11107931	0.93	C	T	0.26	intronic	FGD6					4 altered motifs
12	95591533	rs10859847	0.95	A	G	0.25	intronic	FGD6		NHEK, GM12878	GM06990,SK-N-MC		Pou2f2
12	95592206	rs7309080	0.9	C	T	0.26	intronic	FGD6	GM12878	NHEK, HMEC	GM12865		Foxp1,HNF1
12	95592272	rs7309426	0.91	G	A	0.26	intronic	FGD6	GM12878	NHEK, HMEC	GM12865		LBP-1,YY1,Zbtb3
12	95595892	rs10859848	0.99	G	A	0.24	intronic	FGD6		6 cell types	18 cell types	FOSL1	9 altered motifs
12	95597446	rs68107717	0.92	GGTCA	G	0.26	intronic	FGD6		8 cell types	HRGEC		11 altered motifs

12	95599120	rs2171576	0.92	C	T	0.26	intronic	FGD6		8 cell types			Klf4,Zec
12	95600846	rs10859849	1	C	T	0.24	intronic	FGD6		4 cell types	K562	EGR1	5 altered motifs
12	95600918	rs10859850	1	G	A	0.24	intronic	FGD6		4 cell types	K562,GM12864	EGR1	4 altered motifs
12	95601584	rs10777674	0.93	G	A	0.26	intronic	FGD6		4 cell types			GLI,Zic
12	95602569	rs11107934	1	A	G	0.24	intronic	FGD6		H1	5 cell types		Hoxb8,Zec
**12**	**95604290**	**rs10507047**	**1**	**T**	**C**	**0.24**	**missense**	**FGD6**		**GM12878**			**NF-I**
12	95609305	rs10777676	0.92	C	T	0.26	intronic	FGD6	5 cell types	4 cell types	5 cell types		5 altered motifs
12	95611141	rs6538617	0.9	C	T	0.26	5'-UTR	FGD6	9 cell types			21 bound proteins	14 altered motifs
12	95615631	rs34721136	0.91	CA	C	0.26	intronic	VEZT		4 cell types	BE2_C		8 altered motifs
12	95616085	rs1488643	0.91	G	T	0.26	intronic	VEZT		4 cell types	HMVEC-LLy,HMVEC-dLy-Ad,NHDF-Ad		4 altered motifs
12	95616122	rs1873051	0.91	T	C	0.26	intronic	VEZT		4 cell types	HMVEC-LLy,HMVEC-dLy-Ad,NHDF-Ad		HDAC2,Maf
12	95620706	rs67803117	0.9	T	TA,TT	0.26	intronic	VEZT		NHEK	Fibrobl		
12	95623284	rs6538620	0.91	A	G	0.26	intronic	VEZT		Huvec, K562			
12	95624340	rs11107943	0.98	G	A	0.24	intronic	VEZT		HMEC, Huvec, K562	LNCaP,HMVEC-LLy,HMVEC-dBl-Neo		4 altered motifs
12	95624576	rs10859855	0.91	G	A	0.25	intronic	VEZT		6 cell types	Myometr,HRGEC		RFX5,Roaz
12	95625480	rs6538621	0.91	C	T	0.25	intronic	VEZT		Huvec, K562			BCL
12	95626296	rs4762302	0.98	C	A	0.24	intronic	VEZT			HMEC		4 altered motifs
12	95629171	rs35155695	0.89	G	T	0.26	intronic	VEZT					4 altered motifs
12	95630064	rs9739398	0.9	C	T	0.23	intronic	VEZT					13 altered motifs
12	95630727	rs12830009	0.98	A	T	0.24	intronic	VEZT					5 altered motifs
12	95633378	rs4762152	0.97	T	A	0.24	intronic	VEZT		K562, Huvec	4 cell types		4 altered motifs
12	95633557	rs12369055	0.9	A	G	0.25	intronic	VEZT		K562, Huvec			Mef2
12	95634437	rs11107952	0.97	G	C	0.24	intronic	VEZT			Myometr	BATF	4 altered motifs
12	95635065	rs12815210	0.97	G	T	0.24	intronic	VEZT					SETDB1
12	95635383	rs11107953	0.97	A	T	0.24	intronic	VEZT					
12	95637234	rs1873052	0.9	C	T	0.26	intronic	VEZT		K562			4 altered motifs
12	95637578	rs1873053	0.97	C	A	0.24	intronic	VEZT		K562			TCF11::MafG
12	95641522	rs10859859	0.9	C	T	0.26	intronic	VEZT					Mrg1::Hoxa9,STAT
12	95646229	rs12315763	0.97	T	C	0.24	intronic	VEZT					HMG-IY,PLZF
12	95649270	rs10859861	0.97	C	T	0.24	intronic	VEZT					HNF4,SREBP,Zbtb3
12	95649548	rs12368315	0.97	C	G	0.24	intronic	VEZT					
12	95649762	rs12368359	0.97	G	A	0.24	intronic	VEZT					Ets,LXR,p300
12	95649952	rs11107955	0.97	C	T	0.24	intronic	VEZT					4 altered motifs
12	95651093	rs3751272	0.9	T	G	0.26	intronic	VEZT		H1			Foxj1,Irf
12	95655281	rs11107956	0.97	G	A	0.24	intronic	VEZT		Huvec			7 altered motifs
12	95656522	rs2306885	0.97	A	C	0.24	intronic	VEZT		Huvec	Medullo,pHTE,HA-sp		4 altered motifs
12	95656580	rs144829851	0.9	GAAAAT	G	0.26	intronic	VEZT		Huvec	pHTE,HA-sp	GATA2	18 altered motifs
12	95659865	rs1038653	0.97	C	T	0.24	intronic	VEZT					5 altered motifs

12	95662067	rs10859864	0.97	C	T	0.24	intronic	VEZT					DMRT2,Ets
12	95663552	rs17023867	0.97	T	G	0.24	intronic	VEZT					Nanog,Sox
12	95665912	rs2886933	0.9	T	C	0.26	intronic	VEZT					TEF-1,Zbtb3
12	95666022	rs4762321	0.97	C	T	0.24	intronic	VEZT					HDAC2,Irf,p300
12	95666323	rs4762161	0.9	A	C	0.26	intronic	VEZT					11 altered motifs
12	95666650	rs10859865	0.97	G	A	0.24	intronic	VEZT					
12	95666994	rs12367540	0.97	G	C	0.24	intronic	VEZT					
12	95671324	rs4762327	0.9	G	A	0.26	intronic	VEZT		NHLF, HSMM, Huvec			5 altered motifs
12	95671829	rs10777681	0.97	T	C	0.24	intronic	VEZT		Huvec, NHLF	HMVEC-dLy-Ad		
12	95671904	rs10745720	0.9	A	G	0.26	intronic	VEZT		Huvec, NHLF	HMVEC-dLy-Ad		4 altered motifs
12	95672654	rs11107960	0.97	G	C	0.24	intronic	VEZT					
12	95680901	rs12826627	0.97	A	C	0.24	intronic	VEZT					Arid5a,Hand1
12	95682030	rs10777682	0.97	A	T	0.24	intronic	VEZT					8 altered motifs
12	95683469	rs7137252	0.92	C	T	0.24	intronic	VEZT					4 altered motifs
12	95684817	rs10859867	0.92	G	C	0.24	intronic	VEZT					En-1,STAT,TFII-I
12	95686394	rs4762337	0.85	G	A	0.26	intronic	VEZT					
12	95687428	rs7309226	0.85	A	G	0.26	intronic	VEZT					Pax-5
12	95688475	rs3764046	0.92	A	G	0.24	intronic	VEZT					
12	95688540	rs4762340	0.84	G	A	0.26	intronic	VEZT					OTX,Sox
12	95689093	rs3794321	0.91	C	T	0.24	intronic	VEZT					Sin3Ak-20
12	95690996	rs4762342	0.85	G	A	0.23	intronic	VEZT					Arid5b,Foxp1,HNF1
12	95691539	rs4417384	0.86	A	C	0.23	intronic	VEZT					Mef2
12	95691902	rs6538626	0.8	T	A	0.22	intronic	VEZT		Huvec			10 altered motifs
12	95691905	rs10859868	0.82	T	A	0.22	intronic	VEZT		Huvec			14 altered motifs
12	95695260	rs3185701	0.86	C	T	0.23	3'-UTR	VEZT		Huvec			
12	95695804	rs12298029	0.85	C	A,T	0.23	3'-UTR	VEZT		Huvec			
12	95698006	rs1387047	0.85	C	T	0.23		1.4kb 3' of VEZT		4 cell types	K562		RXRA
12	95699929	rs10777683	0.85	G	T	0.23		2.3kb 5' of MIR331		Huvec			MIZF,Nrf1
12	95703422	rs4762353	0.8	T	C	0.24		275bp 5' of MIR3685					Pax-6,VDR
12	95706225	rs7300457	0.85	T	G	0.26		2.5kb 3' of MIR3685					
12	95706561	rs4762358	0.8	A	G	0.24		2.8kb 3' of MIR3685			Melano		Zfp410
12	95706624	rs10578762	0.8	ATCT T	A	0.24		2.9kb 3' of MIR3685			HCM		Evi-1,Sox
12	95707761	rs10745721	0.8	G	T	0.24		4kb 3' of MIR3685					GR
12	95709315	rs10777684	0.83	G	A	0.23		5.6kb 3' of MIR3685		Huvec	HUVEC		7 altered motifs
12	95710096	rs7308919	0.8	A	T	0.26		6.3kb 3' of MIR3685		Huvec, HepG2	HRCEpiC		4 altered motifs
12	95711526	rs11107974	0.8	G	C	0.26		7.8kb 3' of MIR3685		HepG2, NHLF			4 altered motifs
12	95711543	rs11107975	0.86	C	G	0.24		7.8kb 3' of MIR3685		HepG2, NHLF			NRSF
12	95711682	rs11107976	0.8	A	G	0.26		7.9kb 3' of MIR3685		HepG2, NHLF			BDP1,LUN-1
12	95711876	rs10859871	0.8	A	C	0.26		8.1kb 3' of MIR3685		4 cell types	25 cell types	CEBPB,FOX A1,FOXA2	5 altered motifs
12	95712695	rs4762173	0.8	A	C	0.26		8.9kb 3' of MIR3685		HSMM, NHLF, HepG2			HMG-IY,NF-AT
**16**	**57017292**	**rs2303790**	**1**	**A**	**G**	**0.02**	**missense**	**CETP**	**HepG2**	**GM12878**	**Osteobl,pHTE**		

Supplementary Table 13. RegulomeDB annotation of the four novel genome-wide significant amino acids as well as SNPs in LD ($r^2 > 0.8$) with them.

Chr	Position	SNP-id	SNP-locus	r^2	D'	Hits	Score[a]
**6**	**43970826**	**rs2295334**	***C6orf223***	**1**	**1**	**Motifs|Footprinting|NRSF, Motifs|PWM|NRSF, Motifs|PWM|NRSE, Motifs|Footprinting|NRSE, Chromatin_Structure|FAIRE, Chromatin_Structure|DNase-seq, Protein_Binding|ChIP-seq|FOS, Protein_Binding|ChIP-seq|CTCF**	**2b**
6	43972728	rs2273307	*C6orf223*	0.99	1	Motifs|PWM|SMAD4, Motifs|PWM|MZF1_5-13, Motifs|Footprinting|MZF1_5-13, Motifs|Footprinting|SMAD4, Chromatin_Structure|FAIRE, Chromatin_Structure|DNase-seq, Protein_Binding|ChIP-seq|EBF1	2b
6	43972732	rs2273308	*C6orf223*	0.99	1	Motifs|PWM|SMAD4, Motifs|PWM|MZF1_5-13, Motifs|Footprinting|MZF1_5-13, Motifs|Footprinting|SMAD4, Chromatin_Structure|FAIRE, Chromatin_Structure|DNase-seq, Protein_Binding|ChIP-seq|EBF1	2b
6	43973460	rs9968846	*C6orf223*	0.99	1	Motifs|PWM|Zfp740, Motifs|PWM|UF1H3BETA, Motifs|Footprinting|UF1H3BETA, Chromatin_Structure|DNase-seq, Protein_Binding|ChIP-seq|POLR2A	2b
6	43972624	rs2273306	*C6orf223*	0.99	1	Chromatin_Structure|FAIRE, Chromatin_Structure|DNase-seq, Protein_Binding|ChIP-seq|EBF1	4
6	43974115	rs59822205	*C6orf223*	0.99	1	Chromatin_Structure|DNase-seq, Protein_Binding|ChIP-seq|GATA1, Protein_Binding|ChIP-seq|HNF4A	4
6	43961014	rs73733647	*C6orf223*	0.91	0.95	Motifs|PWM|SREBP, Motifs|Footprinting|SREBP, Motifs|Footprinting|Zic2, Motifs|PWM|Zic2, Chromatin_Structure|DNase-seq	5
6	43963646	rs45584738	*C6orf223*	0.95	0.99	Chromatin_Structure|DNase-seq	5
6	43963767	rs41282646	*C6orf223*	0.95	0.99	Chromatin_Structure|DNase-seq	5
6	43972475	rs60012957	*C6orf223*	0.99	1	Chromatin_Structure|DNase-seq	5
6	43972844	rs2273309	*C6orf223*	0.99	1	Motifs|Footprinting|Pax-3, Motifs|Footprinting|Pax-6, Motifs|PWM|Pax-3, Motifs|PWM|Pax-6, Chromatin_Structure|FAIRE, Chromatin_Structure|DNase-seq	5
6	43973963	rs56118681	*C6orf223*	0.99	1	Motifs|Footprinting|Gfi, Motifs|PWM|Gfi, Motifs|PWM|Gfi1, Motifs|Footprinting|Gfi1, Chromatin_Structure|DNase-seq	5
6	43974835	rs73422500	*C6orf223*	0.99	1	Motifs|Footprinting|mTERF, Motifs|PWM|mTERF, Protein_Binding|ChIP-seq|POLR2A	5
6	43975749	rs73736909	*C6orf223*	0.85	0.95	Chromatin_Structure|DNase-seq	5
6	43964680	rs76098842	*C6orf223*	0.96	0.99	Motifs|PWM|Zfp740, Motifs|PWM|UF1H3BETA	6
**6**	**31842597**	**rs12661281**	***SCL44A4***	**1**	**1**	**Motifs|PWM|Staf**	**6**
12	95611140	rs6538617	*FGD6*	0.9	0.98	Motifs|Footprinting|SP1, Motifs|PWM|Smad3, Motifs|PWM|SP1, Motifs|PWM|AP-2, Motifs|Footprinting|AP-2, Chromatin_Structure|FAIRE, Chromatin_Structure|DNase-seq, Protein_Binding|ChIP-seq|GATA1, Protein_Binding|ChIP-seq|ZNF263, Protein_Binding|ChIP-seq|HMGN3, Protein_Binding|ChIP-seq|HEY1, Protein_Binding|ChIP-seq|EP300, Protein_Binding|ChIP-seq|TFAP2A, Protein_Binding|ChIP-seq|TFAP2C, Protein_Binding|ChIP-seq|RAD21, Protein_Binding|ChIP-seq|NANOG, Protein_Binding|ChIP-seq|TCF4, Protein_Binding|ChIP-seq|CCNT2, Protein_Binding|ChIP-seq|CTBP2, Protein_Binding|ChIP-	2a

						seq|IKZF1, Protein_Binding|ChIP-seq|ETS1, Protein_Binding|ChIP-seq|TBP, Protein_Binding|ChIP-seq|YY1, Protein_Binding|ChIP-seq|POLR2A, Protein_Binding|ChIP-seq|TAF1, Protein_Binding|ChIP-seq|ZBTB7A, Protein_Binding|ChIP-seq|CTCF, Protein_Binding|ChIP-seq|TAF7, Protein_Binding|ChIP-seq|CDX2, Protein_Binding|ChIP-seq|E2F1, Protein_Binding|ChIP-seq|HDAC2	
12	95595891	rs10859848	*FGD6*	0.99	1	Motifs|PWM|Bach1, Motifs|PWM|Jundm2, Chromatin_Structure|FAIRE, Chromatin_Structure|DNase-seq, Protein_Binding|ChIP-seq|FOSL1	3a
12	95600845	rs10859849	*FGD6*	1	1	Motifs|PWM|znf143, Chromatin_Structure|DNase-seq, Protein_Binding|ChIP-seq|EGR1	3a
12	95656521	rs2306885	*FGD6*	0.97	0.99	Motifs|PWM|IRF1, Motifs|PWM|HOXA13, Chromatin_Structure|FAIRE, Chromatin_Structure|DNase-seq, Protein_Binding|ChIP-seq|SPDEF, Protein_Binding|ChIP-seq|AR	3a
12	95600917	rs10859850	*FGD6*	1	1	Chromatin_Structure|DNase-seq, Protein_Binding|ChIP-seq|EGR1	4
12	95633377	rs4762152	*FGD6*	0.97	0.99	Chromatin_Structure|DNase-seq, Protein_Binding|ChIP-seq|GATA1, Protein_Binding|ChIP-seq|HNF4A, Protein_Binding|ChIP-seq|GATA6, Protein_Binding|ChIP-seq|CDX2	4
12	95711875	rs10859871	*FGD6*	0.8	0.93	Chromatin_Structure|FAIRE, Chromatin_Structure|DNase-seq, Protein_Binding|ChIP-seq|HNF4A, Protein_Binding|ChIP-seq|CEBPB, Protein_Binding|ChIP-seq|GATA6, Protein_Binding|ChIP-seq|FOXA2, Protein_Binding|ChIP-seq|FOXA1, Protein_Binding|ChIP-seq|CDX2	4
12	95577883	rs4128507	*FGD6*	0.86	0.97	Motifs|PWM|DMRT2, Motifs|PWM|DMRT4, Motifs|PWM|DMRT3, Chromatin_Structure|DNase-seq	5
12	95591532	rs10859847	*FGD6*	0.95	0.99	Motifs|PWM|Oct-1, Chromatin_Structure|DNase-seq	5
12	95592205	rs7309080	*FGD6*	0.9	0.99	Chromatin_Structure|DNase-seq	5
12	95592271	rs7309426	*FGD6*	0.91	1	Motifs|PWM|CP2/LBP-1c/LSF, Chromatin_Structure|DNase-seq	5
12	95597446	rs68107717	*FGD6*	0.92	1	Chromatin_Structure|DNase-seq	5
12	95599119	rs2171576	*FGD6*	0.92	1	Chromatin_Structure|DNase-seq	5
12	95602568	rs11107934	*FGD6*	1	1	Chromatin_Structure|DNase-seq	5
12	95609304	rs10777676	*FGD6*	0.92	0.99	Motifs|PWM|Cdc5, Motifs|Footprinting|Cdc5, Chromatin_Structure|DNase-seq	5
12	95615631	rs34721136	*FGD6*	0.91	0.99	Motifs|PWM|FOXP1, Motifs|PWM|FOXJ2, Motifs|PWM|Dbx1, Motifs|PWM|BCL6, Motifs|PWM|HNF3beta, Motifs|PWM|HNF3, Motifs|PWM|Oct-1, Chromatin_Structure|DNase-seq	5
12	95616084	rs1488643	*FGD6*	0.91	0.99	Motifs|PWM|TAL1, Chromatin_Structure|DNase-seq	5
12	95616121	rs1873051	*FGD6*	0.91	0.99	Motifs|PWM|Srf, Motifs|PWM|Tcf3, Chromatin_Structure|DNase-seq	5
12	95624339	rs11107943	*FGD6*	0.98	1	Chromatin_Structure|DNase-seq	5
12	95624575	rs10859855	*FGD6*	0.91	0.98	Chromatin_Structure|DNase-seq	5
12	95626295	rs4762302	*FGD6*	0.98	1	Chromatin_Structure|FAIRE, Chromatin_Structure|DNase-seq	5
12	95633556	rs12369055	*FGD6*	0.9	0.97	Motifs|PWM|MEF-2, Protein_Binding|ChIP-seq|HNF4A, Protein_Binding|ChIP-seq|CDX2	5

12	95634436	rs11107952	*FGD6*	0.97	0.99	Motifs|PWM|AFP1, Motifs|Footprinting|AFP1, Protein_Binding|ChIP-seq|BATF	5
12	95637233	rs1873052	*FGD6*	0.9	0.98	Chromatin_Structure|FAIRE, Chromatin_Structure|DNase-seq	5
12	95637577	rs1873053	*FGD6*	0.97	0.99	Chromatin_Structure|DNase-seq	5
12	95641521	rs10859859	*FGD6*	0.9	0.98	Chromatin_Structure|DNase-seq	5
12	95671323	rs4762327	*FGD6*	0.9	0.98	Chromatin_Structure|DNase-seq	5
12	95671828	rs10777681	*FGD6*	0.97	0.99	Chromatin_Structure|DNase-seq	5
12	95671903	rs10745720	*FGD6*	0.9	0.98	Chromatin_Structure|FAIRE, Chromatin_Structure|DNase-seq	5
12	95688474	rs3764046	*FGD6*	0.92	0.96	Protein_Binding|ChIP-seq|RFX3	5
12	95698005	rs1387047	*FGD6*	0.85	0.95	Chromatin_Structure|DNase-seq	5
12	95706560	rs4762358	*FGD6*	0.8	0.9	Chromatin_Structure|FAIRE, Chromatin_Structure|DNase-seq	5
12	95706624	rs10578762	*FGD6*	0.8	0.9	Chromatin_Structure|FAIRE, Chromatin_Structure|DNase-seq	5
12	95710095	rs7308919	*FGD6*	0.8	0.93	Motifs|PWM|Sox18, Motifs|PWM|Sox12, Chromatin_Structure|FAIRE, Chromatin_Structure|DNase-seq	5
12	95559599	rs10859842	*FGD6*	0.87	0.97	Motifs|PWM|FOXP1, Motifs|PWM|Srf, Motifs|PWM|Zfp105, Motifs|PWM|Tcfap2e	6
12	95569636	rs11107925	*FGD6*	0.86	0.98	Motifs|PWM|MZF1, Motifs|PWM|PPARalpha:RXRalpha	6
12	95574830	rs10777670	*FGD6*	0.88	0.99	Motifs|PWM|IRC900814, Chromatin_Structure|FAIRE	6
12	95601583	rs10777674	*FGD6*	0.93	1	Motifs|PWM|GLI, Motifs|PWM|Zic3, Motifs|PWM|GLI1, Motifs|PWM|Zic2, Motifs|PWM|Zic1, Motifs|Footprinting|Zic3	6
12	95630063	rs9739398	*FGD6*	0.9	0.98	Motifs|PWM|Zfp161, Motifs|PWM|Pax5	6
12	95630726	rs12830009	*FGD6*	0.98	0.99	Motifs|PWM|Gata3	6
12	95649951	rs11107955	*FGD6*	0.97	0.99	Motifs|PWM|LUN-1	6
12	95651092	rs3751272	*FGD6*	0.9	0.98	Motifs|PWM|Foxl1, Motifs|PWM|Foxj3	6
12	95663551	rs17023867	*FGD6*	0.97	0.99	Motifs|PWM|SOX9, Motifs|PWM|Sox30	6
12	95666021	rs4762321	*FGD6*	0.97	0.99	Motifs|PWM|Srf, Motifs|PWM|Tcf3, Motifs|PWM|GAF	6
12	95666322	rs4762161	*FGD6*	0.9	0.98	Motifs|PWM|AIRE, Motifs|PWM|aMEF-2	6
12	95682029	rs10777682	*FGD6*	0.97	0.99	Motifs|PWM|Elf3, Chromatin_Structure|FAIRE	6
12	95684816	rs10859867	*FGD6*	0.92	0.96	Motifs|PWM|TFII-I	6
12	95686393	rs4762337	*FGD6*	0.85	0.95	Motifs|PWM|Gata6	6
12	95690995	rs4762342	*FGD6*	0.85	0.95	Motifs|PWM|Gata3, Motifs|PWM|HNF1	6
12	95691538	rs4417384	*FGD6*	0.86	0.96	Motifs|PWM|Gata5	6
12	95691901	rs6538626	*FGD6*	0.8	0.96	Motifs|PWM|FOXP1, Motifs|PWM|Sry, Motifs|PWM|Zfp105, Motifs|PWM|Sox21, Motifs|PWM|Sox14, Motifs|PWM|Oct-1	6

12	95691904	rs10859868	*FGD6*	0.82	0.96	Motifs|PWM|FOXP1, Motifs|PWM|Sry, Motifs|PWM|Zfp105, Motifs|PWM|Sox21, Motifs|PWM|Sox14, Motifs|PWM|Oct-1	6
12	95695259	rs3185701	*FGD6*	0.86	0.96	Motifs|PWM|Zic3	6
12	95699928	rs10777683	*FGD6*	0.85	0.95	Motifs|PWM|MIZF	6
12	95711681	rs11107976	*FGD6*	0.8	0.93	Motifs|PWM|ESE1, Motifs|PWM|LUN-1	6
12	95561194	rs10859843	*FGD6*	0.85	0.97	No data	7
12	95564939	rs7136350	*FGD6*	0.85	0.97	No data	7
12	95575950	rs11107928	*FGD6*	0.93	0.99	No data	7
12	95580817	rs12830425	*FGD6*	0.93	0.99	No data	7
12	95583661	rs4762660	*FGD6*	0.88	0.99	No data	7
12	95587796	rs11107931	*FGD6*	0.93	1	No data	7
**12**	**95604289**	**rs10507047**	***FGD6***	**1**	**1**	**No data**	**7**
12	95620706	rs67803117	*FGD6*	0.9	0.98	No data	7
12	95623283	rs6538620	*FGD6*	0.91	0.99	No data	7
12	95625479	rs6538621	*FGD6*	0.91	0.98	No data	7
12	95629170	rs35155695	*FGD6*	0.89	0.99	No data	7
12	95635064	rs12815210	*FGD6*	0.97	0.99	No data	7
12	95635382	rs11107953	*FGD6*	0.97	0.99	No data	7
12	95646228	rs12315763	*FGD6*	0.97	0.99	No data	7
12	95649269	rs10859861	*FGD6*	0.97	0.99	No data	7
12	95649547	rs12368315	*FGD6*	0.97	0.99	No data	7
12	95649761	rs12368359	*FGD6*	0.97	0.99	No data	7
12	95655280	rs11107956	*FGD6*	0.97	0.99	No data	7
12	95659864	rs1038653	*FGD6*	0.97	0.99	No data	7
12	95662066	rs10859864	*FGD6*	0.97	0.99	No data	7
12	95665911	rs2886933	*FGD6*	0.9	0.98	No data	7
12	95666649	rs10859865	*FGD6*	0.97	0.99	No data	7
12	95666993	rs12367540	*FGD6*	0.97	0.99	No data	7
12	95672653	rs11107960	*FGD6*	0.97	0.99	No data	7
12	95680900	rs12826627	*FGD6*	0.97	0.99	No data	7

12	95683468	rs7137252	*FGD6*	0.92	0.96	No data	7
12	95687427	rs7309226	*FGD6*	0.85	0.95	No data	7
12	95688539	rs4762340	*FGD6*	0.84	0.95	No data	7
12	95689092	rs3794321	*FGD6*	0.91	0.95	No data	7
12	95695803	rs12298029	*FGD6*	0.85	0.95	No data	7
12	95703421	rs4762353	*FGD6*	0.8	0.9	No data	7
12	95706224	rs7300457	*FGD6*	0.85	0.95	No data	7
12	95707760	rs10745721	*FGD6*	0.8	0.9	No data	7
12	95709314	rs10777684	*FGD6*	0.83	0.94	No data	7
12	95711525	rs11107974	*FGD6*	0.8	0.93	No data	7
12	95711542	rs11107975	*FGD6*	0.86	0.93	No data	7
12	95712694	rs4762173	*FGD6*	0.8	0.93	No data	7
**16**	**57017291**	**rs2303790**	***CETP***	**1**	**1**	**Chromatin_Structure|FAIRE, Chromatin_Structure|DNase-seq, Protein_Binding|ChIP-seq|HNF4A**	**4**

r^2 and D' denote pair-wise measures of linkage disequilibrium with the top SNP of each locus (in bold).

[a]RegulomeDB scores indicate the amount of supporting data for regulatory effects at a single coordinate. The lower the scores, the stronger the evidence, with 2b indicating overlapping evidence for transcription factor binding, any motif, DNase footprint and DNase peak at that location, 4 indicating evidence for both transcription factor binding and DNase peak at that location, 5 indicating data for either transcription factor binding or DNase peak, 6 indicating other evidence (e.g. motifs only) and 7 indicating no supporting data for that coordinate.

Supplementary Table 14. Expression quantitative trait results for *SLC44A4* Asp47Val (rs12661281) and *FDG6* Gln257Arg (rs10507047) as well as SNPs in LD ($r^2 > 0.8$) with them

Chr	SNP name	Locus mapping to SNP	r^2	D'	eQTL hit	eQTL Chr	FDR	p value	SNP type	Allele assessed	Overall Z score
**6**	**rs12661281**	*SLC44A4*	**1**	**1**	***HSPA1B***	**6**	**<0.001**	**3.84E-34**	**T/A**	**A**	**12.18**
**6**	**rs12661281**	*SLC44A4*	**1**	**1**	***CSNK2B***	**6**	**<0.001**	**2.54E-24**	**T/A**	**A**	**-10.18**
**6**	**rs12661281**	*SLC44A4*	**1**	**1**	***HSPA1L***	**6**	**0.030**	**7.41E-05**	**T/A**	**A**	**3.96**
**6**	**rs12661281**	*SLC44A4*	**1**	**1**	***BAT3***	**6**	**0.13**	**4.05E-04**	**T/A**	**A**	**3.54**
**6**	**rs12661281**	*SLC44A4*	**1**	**1**	***LY6G5C***	**6**	**0.19**	**7.16E-04**	**T/A**	**A**	**3.38**
12	rs11107925	*FGD6*	0.86	0.98	*NDUFA12*	12	<0.001	4.39E-40	G/C	C	-13.25
12	rs4128507	*FGD6*	0.86	0.97	*NDUFA12*	12	<0.001	8.80E-38	T/C	C	-12.85
12	rs7309080	*FGD6*	0.9	0.99	*NDUFA12*	12	<0.001	1.24E-37	T/C	T	-12.82
12	rs10777670	*FGD6*	0.88	0.99	*NDUFA12*	12	<0.001	1.25E-37	G/A	A	-12.82
12	rs10745720	*FGD6*	0.9	0.98	*VEZT*	12	<0.001	3.11E-31	G/A	G	11.62
12	rs4417384	*FGD6*	0.86	0.96	*VEZT*	12	<0.001	3.59E-31	C/A	C	11.61
12	rs10777684	*FGD6*	0.83	0.94	*VEZT*	12	<0.001	3.97E-31	G/A	A	11.60
12	rs1488643	*FGD6*	0.91	0.99	*VEZT*	12	<0.001	1.97E-30	T/G	T	11.47
12	rs10777674	*FGD6*	0.93	1	*VEZT*	12	<0.001	2.35E-30	G/A	A	11.45
12	rs10777676	*FGD6*	0.92	0.99	*VEZT*	12	<0.001	2.54E-30	T/C	T	11.44
12	rs4762340	*FGD6*	0.84	0.95	*VEZT*	12	<0.001	1.71E-27	G/A	A	10.86
12	rs12298029	*FGD6*	0.85	0.95	*VEZT*	12	<0.001	1.88E-27	C/A	A	10.86
12	rs1387047	*FGD6*	0.85	0.95	*VEZT*	12	<0.001	1.88E-27	T/C	T	10.86
12	rs10777683	*FGD6*	0.85	0.95	*VEZT*	12	<0.001	2.40E-27	T/G	T	10.83
12	rs7308919	*FGD6*	0.8	0.93	*VEZT*	12	<0.001	2.78E-27	T/A	T	10.82
12	rs10859871	*FGD6*	0.8	0.93	*VEZT*	12	<0.001	2.78E-27	C/A	C	10.82
12	rs10859855	*FGD6*	0.91	0.98	*VEZT*	12	<0.001	3.45E-24	G/A	A	10.15
12	rs10777674	*FGD6*	0.93	1	*NDUFA12*	12	<0.001	3.30E-22	G/A	A	-9.69
12	rs10777676	*FGD6*	0.92	0.99	*NDUFA12*	12	<0.001	4.93E-22	T/C	T	-9.65
12	rs1038653	*FGD6*	0.97	0.99	*VEZT*	12	<0.001	5.32E-22	T/C	T	9.64
12	rs4762321	*FGD6*	0.97	0.99	*VEZT*	12	<0.001	5.32E-22	T/C	T	9.64

12	rs12367540	*FGD6*	0.97	0.99	*VEZT*	12	<0.001	5.32E-22	G/C	C	9.64
12	rs10777681	*FGD6*	0.97	0.99	*VEZT*	12	<0.001	5.32E-22	T/C	C	9.64
12	rs1873053	*FGD6*	0.97	0.99	*VEZT*	12	<0.001	5.92E-22	C/A	A	9.63
12	rs12315763	*FGD6*	0.97	0.99	*VEZT*	12	<0.001	5.92E-22	T/C	C	9.63
12	rs12368315	*FGD6*	0.97	0.99	*VEZT*	12	<0.001	5.92E-22	G/C	G	9.63
12	rs12368359	*FGD6*	0.97	0.99	*VEZT*	12	<0.001	5.92E-22	G/A	A	9.63
12	rs2306885	*FGD6*	0.97	0.99	*VEZT*	12	<0.001	5.92E-22	C/A	C	9.63
12	rs1488643	*FGD6*	0.91	0.99	*NDUFA12*	12	<0.001	7.40E-22	T/G	T	-9.61
12	rs11107925	*FGD6*	0.86	0.98	*VEZT*	12	<0.001	1.73E-21	G/C	C	9.52
12	rs11107934	*FGD6*	1	1	*VEZT*	12	<0.001	2.12E-21	G/A	G	9.50
**12**	**rs10507047**	*FGD6*	**1**	**1**	***VEZT***	**12**	**<0.001**	**2.82E-21**	**T/C**	**C**	**9.47**
12	rs10859850	*FGD6*	1	1	*VEZT*	12	<0.001	3.11E-21	G/A	A	9.46
12	rs7309080	*FGD6*	0.9	0.99	*VEZT*	12	<0.001	6.62E-21	T/C	T	9.38
12	rs4128507	*FGD6*	0.86	0.97	*VEZT*	12	<0.001	3.51E-20	T/C	C	9.20
12	rs10777670	*FGD6*	0.88	0.99	*VEZT*	12	<0.001	7.43E-20	G/A	A	9.12
12	rs10859855	*FGD6*	0.91	0.98	*NDUFA12*	12	<0.001	8.86E-15	G/A	A	-7.75
12	rs12826627	*FGD6*	0.97	0.99	*VEZT*	12	<0.001	1.98E-14	C/A	C	7.65
12	rs10859847	*FGD6*	0.95	0.99	*VEZT*	12	<0.001	1.51E-12	G/A	G	7.07
12	rs11107928	*FGD6*	0.93	0.99	*VEZT*	12	<0.001	3.50E-12	C/A	A	6.96
12	rs3185701	*FGD6*	0.86	0.96	*VEZT*	12	<0.001	5.07E-12	T/C	T	6.90
12	rs10859847	*FGD6*	0.95	0.99	*NDUFA12*	12	<0.001	1.01E-09	G/A	G	-6.11
12	rs11107928	*FGD6*	0.93	0.99	*NDUFA12*	12	<0.001	1.39E-09	C/A	A	-6.06

Chr, chromosome; FDR, false discovery rate

Supplementary Table 15. Study power as a function of minor allele frequency and odds ratio for genetic markers **A**) surpassing $P < 1 \times 10^{-4}$ in the discovery collection of 2,119 exudative AMD cases and 5,691 controls so that they could be brought forward for further testing in the replication stage and **B**) surpassing $P < 5 \times 10^{-8}$ in the combined meta-analysis of 6,345 exudative AMD cases and 15,980 controls. Conditions yielding ≥80% power are shaded in light blue.

A

Minor allele frequency	Odds ratio 1.15	1.20	1.25	1.30	1.35	1.40	1.45	1.50	1.55	1.60
**0.05**	1.8%	6.4%	17%	34.7%	56.3%	75.8%	89.2%	96.1%	98.9%	>99%
**0.10**	7.5%	25.7%	54.6%	80.9%	94.7%	>99%	>99%	>99%	>99%	>99%
**0.15**	16.2%	47.4%	79.9%	95.7%	>99%	>99%	>99%	>99%	>99%	>99%
**0.20**	25.6%	64.2%	91.3%	>99%	>99%	>99%	>99%	>99%	>99%	>99%
**0.25**	34.2%	75.3%	96%	>99%	>99%	>99%	>99%	>99%	>99%	>99%
**0.30**	41.1%	82.1%	97.9%	>99%	>99%	>99%	>99%	>99%	>99%	>99%
**0.35**	46.2%	86%	98.7%	>99%	>99%	>99%	>99%	>99%	>99%	>99%
**0.40**	49.5%	88.1%	99%	>99%	>99%	>99%	>99%	>99%	>99%	>99%
**0.45**	51.1%	88.9%	>99%	>99%	>99%	>99%	>99%	>99%	>99%	>99%
**0.50**	51.1%	88.8%	>99%	>99%	>99%	>99%	>99%	>99%	>99%	>99%

B

Minor allele frequency	Odds ratio 1.15	1.20	1.25	1.30	1.35	1.40	1.45	1.50	1.55	1.60
**0.05**	0.9%	8.2%	33.7%	70.4%	93%	>99%	>99%	>99%	>99%	>99%
**0.10**	10.6%	53.7%	92.1%	>99%	>99%	>99%	>99%	>99%	>99%	>99%
**0.15**	31.6%	86.5%	>99%	>99%	>99%	>99%	>99%	>99%	>99%	>99%
**0.20**	53.6%	96.7%	>99%	>99%	>99%	>99%	>99%	>99%	>99%	>99%
**0.25**	69.8%	>99%	>99%	>99%	>99%	>99%	>99%	>99%	>99%	>99%
**0.30**	79.8%	>99%	>99%	>99%	>99%	>99%	>99%	>99%	>99%	>99%
**0.35**	85.5%	>99%	>99%	>99%	>99%	>99%	>99%	>99%	>99%	>99%
**0.40**	88.5%	>99%	>99%	>99%	>99%	>99%	>99%	>99%	>99%	>99%
**0.45**	89.8%	>99%	>99%	>99%	>99%	>99%	>99%	>99%	>99%	>99%
**0.50**	89.7%	>99%	>99%	>99%	>99%	>99%	>99%	>99%	>99%	>99%

Supplementary Methods

Discovery Cohorts

Singapore

Cases: The exudative age-related macular degeneration (AMD) cases were recruited from the retinal clinics of three major public hospitals with tertiary eye care in Singapore, including the Singapore National Eye Center, National University Health System, and Tan Tock Seng Hospital, between September 2007 and April 2011.[11] For this study, we only included patients of Chinese ethnicity. Each AMD patient was examined according to a standardized protocol to capture clinical, imaging and functional data of the disease. The standardized examination procedure was derived in part from the Singapore Chinese Eye Study (SCES).[12] Patients underwent a comprehensive ocular examination, including dilated fundus examination, fundus angiography with fluorescein and indocyanine green (ICG) using a fundus camera (TRC-50X/IMAGEnet 2000,Topcon, Tokyo, Japan) or confocal scanning laser ophthalmoscope (Spectralis, Heidelberg Engineering, Heidelberg, Germany), and optical coherence tomography (OCT) (Cirrus, Carl Zeiss Meditec, Dublin, California, USA). AMD was graded based on retinal photos following the Wisconsin Age-Related Maculopathy Grading System.[13] For each sample, 10-20 mL of venous blood was collected for DNA extraction for genotyping purposes.

Controls: The controls comprised participants from the Singapore Chinese Eye Study (SCES), without any clinical sign of AMD. The SCES is a population-based cross-sectional study of eye diseases in Chinese adults 40 years of age or older residing in the south-western part of Singapore[12] (see more detailed description about SCES in the “Population-based Cohorts for Analysis of the Association between D442G and HDL” section).

Hong Kong

Cases: All study subjects were Southern Han Chinese of Guangdong descent recruited at the Hong Kong Eye Hospital and the Prince of Wales Hospital Eye Center, Hong Kong. All patients were given complete ophthalmic examinations, including best-corrected visual acuity measurement, ocular tonometry, slit-lamp biomicroscopy, and color fundus photographs. Fluorescein angiography and high-speed confocal ICG angiography (Specrtalis, Heidelberg Engineering, Heidelberg, Germany) were performed in all patients to confirm the diagnosis.

Clinical diagnosis and categorization of AMD followed the Age-Related Eye Disease Study (AREDS) criteria.[14] All AMD cases had exudative AMD in at least one eye. Subjects with any eye with other causes of choroidal neovascularization, such as myopic maculopathy were excluded.

Controls: The controls were of Han Chinese of Guangdong descent, enrolled as previously described.[1] Venous blood was collected from patients to extract DNA.

Japan

Cases: All exudative AMD cases were of Japanese descent recruited from the Department of Ophthalmology at Kyoto University Hospital, Fukushima Medical University Hospital, and the Kobe City Medical Center General Hospital. All patients underwent a comprehensive ophthalmic examination, including determination of best-corrected visual acuity, intraocular pressure measurement, indirect ophthalmoscopy, slit-lamp biomicroscopy with a contact lens, and OCT examination (Spectralis, Heidelberg Engineering, Heidelberg, Germany; and/or Cirrus, Carl Zeiss Meditec, Dublin, California, USA). After fundus photographs were taken, fluorescein angiography and ICG angiography were performed on each patient, using a confocal laser scanning system (Heidelberg Retina Angiography II, Heidelberg Engineering, Heidelberg, Germany). Venous blood was collected to extract DNA for genotyping.

Controls: The control subjects were cataract patients without signs of AMD in both eyes and were recruited from the Department of Ophthalmology at Kyoto University Hospital, the Ozaki Eye Hospital, Mizoguchi Eye Clinic, the Japanese Red Cross Otsu Hospital, and Nagahama City Hospital.

Replication Cohorts

Korea

Cases: All study subjects were unrelated and of native Korean descent, aged 50 years or older, recruited from 6 University Hospital-based tertiary retinal care centers, including the Seoul National University Bundang Hospital in Seongnam; Seoul National University Hospital in Seoul; Kyungpook National University Hospital and Yeungnam University Hospital in Daegu;

and Kosin University Hospital and Busan Paik Hospital in Busan, Korea. Each AMD patient was examined according to a standardized protocol to capture clinical, imaging and functional data of the disease. Patients underwent a comprehensive ocular examination, including dilated fundus examination, fundus photography, OCT (Spectralis, Heidelberg Engineering, Heidelberg, Germany; and/or Cirrus, Carl Zeiss Meditec, Dublin, California, USA) fundus fluorescein angiography and ICG angiography (Heidelberg Retina Angiography, Heidelberg Engineering, Heidelberg, Germany). Based on clinical and ocular examination results, patients were categorized according to the AREDS classification system.[14] Late AMD was diagnosed and evaluated with fundus photograph, fluorescein and ICG angiography, and was categorized into typical neovascular AMD, polypoidal choroidal vasculopathy and geographic atrophy according examination findings. Subjects with geographic atrophy were excluded from the current study. A 10-20 ml sample of venous blood was collected to extract DNA for genotyping.

Controls: Subjects without any sign of AMD were enrolled as controls. They had no drusen and pigment abnormalities in the fundus photograph and/or optical coherence tomography.

Japan

Cases: Subjects with exudative AMD were recruited from Department of Ophthalmology at Saitama Medical University Hospital, Department of Ophthalmology at Yamanashi University Hospital, and Department of Ophthalmology at Kobe University Hospital, following similar protocols as used in the Japan discovery cohort.

Controls: Controls were drawn from two resources. First, we used DNA samples from 929 subjects randomly selected from the Japanese Pharma SNP Consortium. This group has been used for previous genomic studies and is regarded as being representative of the general Japanese population without any history of ocular disease. Second, we used 3,248 participants in the Nagahama Prospective Genome Cohort for the Comprehensive Human Bioscience (the Nagahama Study, see more detailed description about SCES in the “Population-based Cohorts for Analysis of the Association between D442G and HDL” section).

Guangdong, China

Cases: All study subjects were Southern Han Chinese of Guangdong descent recruited at the Zhongshan Ophthalmic Center and Joint Shantou International Eye Center. Cases with

exudative AMD underwent similar ocular examination as for the discovery collection in Hong Kong. The same recruitment criteria and were applied as for the discovery collection.

Controls: Controls were population-based controls of Southern Han Chinese Guangdong descent recruited at the Sun Yet Sen University Cancer Centre, as previously described.[15]

Sichuan, China

Cases: Patients with exudative AMD and age-matched controls were recruited from the ophthalmology clinic at Sichuan Provincial People's Hospital. All exudative AMD cases patients were newly diagnosed and treatment-naïve. They all underwent bilateral ophthalmic examinations including visual acuity measurements, slit-lamp biomicroscopy, fundoscopy, color fundus photography, fluorescein angiography and ICG angiography. Diagnosis was based on the worst eye, but cases with comorbidity of any other retinal or choroidal disease in one or both eyes were excluded. Patients with other choroidal neovascularization due to pathologic myopia, angioid streaks, multifocal choroiditis and punctate inner choroidopathy were excluded.

Controls: All control subjects were aged at least 50 years and underwent ophthalmic examinations including visual acuity measurements, slit-lamp biomicroscopy, ophthalmoscopy and color fundus photography. Those with macular degeneration of any cause, macular changes (such as drusen or pigment abnormalities), or media opacities preventing the clear visualization of the macula were excluded from the study.

Beijing, China

Cases: All study subjects were Northern Han Chinese recruited at the Department of Ophthalmology, Peking University People's Hospital. All subjects received a standard ophthalmic examination, including visual acuity measurement, slit-lamp biomicroscopy, and dilated fundus examination that performed by a retinal specialist. All cases underwent fluorescein angiography, OCT, and ICG angiograms with Heidelberg Retina Angiography II (Heidelberg Engineering, Heidelberg, Germany). Clinical diagnosis and categorization of AMD followed the AREDS criteria.[14] All AMD patients had exudative AMD in at least one eye. Eyes with other macular abnormalities, such as pathologic myopia, idiopathic choroidal neovascularization, presumed ocular histoplasmosis, angioid streaks, and other secondary choroidal neovascularization, were excluded.

Controls: Normal controls were defined as no clinical evidence of any AMD signs in either eye or any other eye diseases, except mild age-related cataract. Subjects with severe cataracts were excluded from the study.

Population-based Cohorts for Analysis of the Association between D442G and HDL

Singapore Chinese Eye Study

The SCES is a population-based cross-sectional study of eye diseases in Chinese adults 40 years of age or older residing in Singapore as described in the Singapore Discovery Cohort section. The methodology of the SCES study has been described in detail elsewhere.[12] Between 2009 and 2011, 3,353 (72.8%) of 4,605 eligible individuals underwent a comprehensive ophthalmologic examination, including dilated fundus examination and retinal photography. A digital fundus camera (Canon CR-DGi with 10D SLR digital camera back; Canon, Tokyo, Japan) was used to capture color photographs of retina of each eye after pupil dilation. AMD was graded based on retinal photos following the Wisconsin Age-Related Maculopathy Grading System.[13] Venous blood was collected and stored for DNA extraction. All participants had an interview (smoking status, use of lipid-lowering medication, etc.), systemic examination, and laboratory investigations using standard laboratory techniques, including measurements of serum lipid profiles. Genome-wide genotyping was done on 2,504 subjects using Illumina Human610-Quad (N =1889) or HumanOmniExpress (N = 615) BeadChips as described previously, followed by strict genotype quality controls.[16]

Singapore Prospective Study Program

Samples of Singapore Prospective Study Program (SP2) were from a revisit of 4 previously conducted population-based surveys carried out in Singapore, including the Thyroid and Heart Study1982-1984,[17] the National Health Survey 1992,[18] the National University of Singapore Heart Study 1993-1995,[19] and the National Health Survey 1998.[20] These studies comprise random samplings of individuals from the Singapore population aged 24 to 95 years, with disproportionate sampling stratified by ethnicity to ensure sufficient sample size in minority ethnic groups. The details of study design, subject recruitments and clinical protocols were described previously.[21, 22] In brief, 5,157 subjects attended a comprehensive clinic examination at the Singapore Eye Research Institute that included systemic and ocular examination, retinal

photography, and laboratory investigations. Venous blood samples were analyzed for lipid profiles at the National University Hospital Reference Laboratory for using an automated autoanalyzer (ADVIA 2400; Bayer Diagnostics, New York).

The genotyping for SP2 involved individuals of Chinese descent only.[23] Of the 2,867 blood-derived DNA samples, 392 samples were genotyped on the HumanHap 550v3, 1,459 samples on the 610-Quad, 817 samples on the 1M-Duov3, 191 samples on both 550v3 and 1M-Duov3, and 8 samples on both 610-Quad and 1M-Duov3. For the samples that were genotyped on two platforms, we used the genotypes from the denser platform in our study.

Nagahama Prospective Genome Cohort for the Comprehensive Human Bioscience

The Nagahama Prospective Genome Cohort for the Comprehensive Human Bioscience (the Nagahama Study) is a community-based prospective cohort study that aims to determine the prevalence and risk factors of various diseases in a community. The details of study design and methodology have been described elsewhere.[24] In brief, residents of Nagahama City who satisfied the following criteria were recruited as participants and were examined between November 2008 and November 2010: 1) age ≥30 and ≤74 years; 2) ability to participate on one's own; 3) no significant problems communicating in Japanese; 4) no current serious diseases/symptoms or health issues; and 5) voluntarily decided to participate in this study. A total of 9,804 Japanese individuals participated in the Nagahama Study. All participants underwent fundus photography using a digital retinal camera (CR-DG10; Canon, Tokyo, Japan) in a dark room.[24] The fundus photos were graded for the individuals aged ≥50 years (N = 6,118) according to the simplified severity scale for AMD in the AREDS Study.[25] Information on smoking status and anti-lipid medication and was obtained via a self-reported questionnaire. Smoking status was converted to Brinkman index.

Genomic DNA was extracted from peripheral blood samples by phenol-chloroform method. Of the participants, 3,712 individuals were genome-scanned using HumanHap610K Quad Arrays, HumanOmni2.5M Arrays, and/or HumanExome Arrays (Illumina Inc., San Diego, California, USA). After our standard quality control, genomic imputation was performed on 192 participants’ data that had been genotyped by every platform. Finally, the data that consists of 1,756,611 SNPs of 3,248 individuals were fixed. All study procedures were approved by the ethics committee of Kyoto University Graduate School of Medicine.

Coronary Heart Disease Case-Controls Collection in Singapore

The Singapore Chinese Health Study (SCHS) is a cohort study of 63,257 Singaporean Chinese men and women of the Hokkien or Cantonese dialect group aged 45-74 years and residing in public housing estates (where 86% of Singaporeans live). Recruitment and the assessment of baseline diet and other characteristics through in-person interviews in the participant’s home took place from 1993 to 1998 (response rate 85%). Non-fasting venous blood was collected in 28,439 participants between 2000 and 2005. Less than 1% of study subjects are lost to follow-up. The current data is from a nested case-control study of coronary heart disease within SCHS participants without cardiovascular disease at the time of blood collection.

Cases had fatal coronary heart disease (CHD) or non-fatal myocardial infarction identified through the Singapore Registry of Births and Deaths and the Hospital Discharge Database respectively. For all non-fatal cases we retrieved medical records for review by a cardiologist and only included those that had confirmed MI using the criteria of the Multi-Ethnic Study of Atherosclerosis. Cases of fatal CHD were only included if there was prior evidence of CHD based on the questionnaire data or the Hospital Discharge Database. Controls were participants who were alive and free of CHD at the time of the diagnosis or death of the index cases and matched (2 to 1) for sex, dialect group, year of birth, year of recruitment and date of blood collection.

Supplementary References

1. Vithana, E. N., *et al.* Genome-wide association analyses identify three new susceptibility loci for primary angle closure glaucoma. *Nat. Genet.* **44**, 1142-1146 (2012).
2. Cornes, B. K., *et al.* Identification of four novel variants that influence central corneal thickness in multi-ethnic Asian populations. *Hum. Mol. Genet.* **21**, 437-445 (2012).
3. Yu, Y., *et al.* Common variants near FRK/COL10A1 and VEGFA are associated with advanced age-related macular degeneration. *Hum Mol Genet* **20**, 3699-3709 (2011).
4. Fritsche, L. G. , *et al.* Seven new loci associated with age-related macular degeneration. *Nat. Genet.* **45**, 433-439, 439e431-432 (2013).
5. Takeuchi, F., *et al.* Association of genetic variants influencing lipid levels with coronary artery disease in Japanese individuals. *PLoS One* **7**, e46385 (2012).
6. Zhan, X., *et al.* Identification of a rare coding variant in complement 3 associated with age-related macular degeneration. *Nat. Genet.* **45**, 1375-1379 (2013).
7. Seddon, J. M., *et al.* Rare variants in CFI, C3 and C9 are associated with high risk of advanced age-related macular degeneration. *Nat. Genet.* **45**, 1366-1370 (2013).
8. Helgason, H., *et al.* A rare nonsynonymous sequence variant in C3 is associated with high risk of age-related macular degeneration. *Nat. Genet.* **45**, 1371-1374 (2013).
9. Kathiresan, S., *et al.* Common variants at 30 loci contribute to polygenic dyslipidemia. *Nat. Genet.* **41**, 56-65 (2009).
10. Kathiresan, S., *et al.* Six new loci associated with blood low-density lipoprotein cholesterol, high-density lipoprotein cholesterol or triglycerides in humans. *Nat. Genet.* **40**, 189-197 (2008).
11. Cheung, C. M., *et al.* Asian age-related macular degeneration phenotyping study: rationale, design and protocol of a prospective cohort study. *Clin. Experiment. Ophthalmol.* **40**, 727-735 (2012).
12. Lavanya, R., *et al.* Methodology of the Singapore Indian Chinese Cohort (SICC) eye study: quantifying ethnic variations in the epidemiology of eye diseases in Asians. *Ophthalmic Epidemiol.* **16**, 325-336 (2009).
13. Klein, R., Davis, M. D., Magli, Y. L., Segal, P, Klein, B. E., Hubbard, L. The Wisconsin age-related maculopathy grading system. *Ophthalmology* **98**, 1128-1134 (1991).
14. Davis, M. D., *et al.* The Age-Related Eye Disease Study severity scale for age-related macular degeneration: AREDS Report No. 17. *Arch. Ophthalmol.* **123**, 1484-1498 (2005).
15. Bei, J. X., *et al.* A genome-wide association study of nasopharyngeal carcinoma identifies three new susceptibility loci. *Nat. Genet.* **42**, 599-603 (2010).
16. Cornes, B. K., *et al.* Identification of four novel variants that influence central corneal thickness in multi-ethnic Asian populations. *Hum. Mol. Genet.* **21**, 437-445 (2012).
17. Hughes, K., *et al.* Cardiovascular diseases in Chinese, Malays, and Indians in Singapore. II. Differences in risk factor levels. *J. Epidemiol. Community Health.* **44**, 29-35 (1990).
18. Tan, C. E., Emmanuel, S. C., Tan, B. Y., Jacob E. Prevalence of diabetes and ethnic differences in cardiovascular risk factors. The 1992 Singapore National Health Survey. *Diabetes Care* **22**, 241-247 (1999).
19. Hughes, K., Aw, T. C., Kuperan, P., Choo, M. Central obesity, insulin resistance, syndrome X, lipoprotein(a), and cardiovascular risk in Indians, Malays, and Chinese in Singapore. *J. Epidemiol. Community. Health* **51**, 394-399 (1997).

20. Cutter, J., Tan, B. Y., Chew, S. K. Levels of cardiovascular disease risk factors in Singapore following a national intervention programme. *Bull. World Health Organ.* **79**, 908-915 (2001).
21. Sabanayagam, C., *et al*. Retinal arteriolar narrowing increases the likelihood of chronic kidney disease in hypertension. *J. Hypertens.* **27**, 2209-2217 (2009).
22. Cheung, C. M.*, et al.* Prevalence of and risk factors for age-related macular degeneration in a multiethnic Asian cohort. *Arch. Ophthalmol.* **130**, 480-486 (2012).
23. Sim, X*, et al.* Transferability of type 2 diabetes implicated loci in multi-ethnic cohorts from Southeast Asia. *PLoS Genet* **7**, e1001363 (2011).
24. Nakata, I*, et al.* Prevalence and characteristics of age-related macular degeneration in the Japanese population: the nagahama study. *Am. J. Ophthalmol.* **156**, 1002-1009 e1002 (2013).
25. Ferris, F. L.*, et al.* A simplified severity scale for age-related macular degeneration: AREDS Report No. 18. *Arch. Ophthalmol.* **123**, 1570-1574 (2005).
